# Precision targeting of β-catenin induces tumor reprogramming and immunity in hepatocellular cancers

**DOI:** 10.21203/rs.3.rs-5494074/v1

**Published:** 2024-12-12

**Authors:** Brandon M. Lehrich, Evan R. Delgado, Tyler M. Yasaka, Silvia Liu, Catherine Cao, Yuqing Liu, Mohammad Taheri, Xiangnan Guan, Hartmut Koeppen, Sucha Singh, Jia-Jun Liu, Anya Singh-Varma, Yekaterina Krutsenko, Minakshi Poddar, T. Kevin Hitchens, Lesley M. Foley, Binyong Liang, Alex Rialdi, Ravi P. Rai, Panari Patel, Madeline Riley, Aaron Bell, Reben Raeman, Tulin Dadali, Jason J. Luke, Ernesto Guccione, Mo R. Ebrahimkhani, Amaia Lujambio, Xin Chen, Martin Maier, Yulei Wang, Wendy Broom, Junyan Tao, Satdarshan P. Monga

**Affiliations:** 1 Department of Pharmacology and Chemical Biology, University of Pittsburgh School of Medicine, Pittsburgh, PA, USA; 2 Pittsburgh Liver Research Center, University of Pittsburgh and University of Pittsburgh Medical Center, Pittsburgh, PA, USA; 3 Medical Scientist Training Program, University of Pittsburgh, Pittsburgh, Pennsylvania, USA; 4 Department of Pathology, University of Pittsburgh School of Medicine, Pittsburgh, PA, USA; 5 Translational Medicine, Genentech Inc., San Francisco, CA, USA; 6 University of Pittsburgh School of Medicine, Pittsburgh, Pennsylvania, USA; 7 Hepatic Surgery Center, Department of Surgery, Tongji Hospital, Tongji Medical College, Huazhong University of Science and Technology, Wuhan, China; 8 Department of Oncological Sciences, Icahn School of Medicine at Mount Sinai, New York, NY; 9 Alnylam Pharmaceuticals, Boston, MA, USA; 10 UPMC Hillman Cancer Center and University of Pittsburgh, Pittsburgh, PA; 11 Cancer Biology Program, University of Hawaii Cancer Center, Honolulu, HI, USA; 12 Division of Gastroenterology, Hepatology and Nutrition, Department of Medicine, University of Pittsburgh School of Medicine, Pittsburgh, PA, USA

**Keywords:** hepatocellular carcinoma, Wnt, β-catenin, immunotherapy, molecular therapy, single cell, spatial transcriptomics, precision medicine

## Abstract

First-line immune checkpoint inhibitor (ICI) combinations show responses in subsets of hepatocellular carcinoma (HCC) patients. Nearly half of HCCs are Wnt-active with mutations in *CTNNB1* (encoding for β-catenin), *AXIN1/2*, or *APC*, and demonstrate limited benefit to ICI due to an immune excluded tumor microenvironment. We show significant tumor responses in multiple β-catenin-mutated immunocompetent HCC models to a novel siRNA encapsulated in lipid nanoparticle targeting *CTNNB1* (LNP-CTNNB1). Both single-cell and spatial transcriptomics revealed cellular and zonal reprogramming of *CTNNB1*-mutated tumors, along with activation of immune regulatory transcription factors IRF2 and POU2F1, re-engaged type I/II interferon signaling, and alterations in both innate and adaptive immune responses upon β-catenin suppression with LNP-CTNNB1. Moreover, LNP-CTNNB1 synergized with ICI in advanced-stage disease through orchestrating enhanced recruitment of cytotoxic T cell aggregates. Lastly, *CTNNB1*-mutated patients treated with atezolizumab plus bevacizumab combination had decreased presence of lymphoid aggregates, which were prognostic for response and survival. In conclusion, LNP-CTNNB1 is efficacious as monotherapy and in combination with ICI in *CTNNB1*-mutated HCCs through impacting tumor cell intrinsic signaling and remodeling global immune surveillance, providing rationale for clinical investigations.

## INTRODUCTION

Hepatocellular carcinoma (HCC) is the third leading cause of cancer-related death globally.^1^ Despite the shift in therapeutic management of advanced disease over the last five years from multi-tyrosine kinase inhibitors (TKIs) (e.g., sorafenib) to immunotherapy with immune-checkpoint inhibitor (ICI) combinations (e.g., atezolizumab plus bevacizumab), objective response rates (ORRs) remain low at ~30% with overall survival <2 years.^2–5^ Preclinical and clinical studies investigating molecular correlates of ICI response have yielded novel insights into potential mechanisms of resistance, including but not limited to immune exclusion, with Wnt/β-catenin activation contributing to this phenotype.^6–8^ Wnt/β-catenin pathway activity is observed in up to 50% of tumors from patients with HCC, with mutations mostly occurring in *CTNNB1* (26–37%), *AXIN1/2* (8–10%), and *APC* (3–5%).^9–12^ Gain-of-function (GOF) mutations in *CTNNB1* (encoding for β-catenin) are one of the major trunk mutational events in HCC and occur mostly as missense mutations in exon 3 at serine and threonine residues or the ubiquitination destruction motif, which interfere with its degradation, leading to constitutive β-catenin activation and target gene transcription.^13,14^ Patients with *CTNNB1*-mutated HCC have upregulation of known Wnt/β-catenin target genes, including *GLUL, AXIN2, LGR5*, and *TBX3*.^11^ In fact, glutamine synthetase (GS; encoded by GLUL) immunohistochemistry is used as a biomarker for patients with CTNNB1-mutated HCC.^15^ However, targeting these downstream Wnt target genes has revealed novel negative feedback loops in the Wnt/β-catenin oncogenic circuit,^16,17^ necessitating the need to focus on targeting β-catenin directly for precision therapy.

Despite improved molecular stratification of HCC over the last decade, with recognition of Wnt/β-catenin driven tumors overlapping with Hoshida S3^18^ or Boyault G5/G6 subclasses^19^, these different molecular stratifications have not yielded prognostic implications due to a lack of clinically approved targeted or biomarker-driven precision therapeutics. β-catenin has traditionally been an “undruggable” target, despite preclinical studies elucidating the molecular and metabolic addiction to β-catenin oncogenic signaling in *CTNNB1*-mutated HCC.^20–23^ Thus, β-catenin is a prime target for precision therapy. Advances in RNAi technology over the last two decades have resulted in multiple approved RNAi therapies,^24^ and RNAi-mediated gene silencing has proven to be an excellent tool for targeting the traditionally “undruggable”, especially in hepatic tissue.

In the current study, we investigate the relevance of RNAi-mediated β-catenin inhibition in patient-derived *CTNNB1*-mutated HCC organoids and multiple humanized mouse models of *CTNNB1*-mutated HCC at different treatment windows and elucidate the underlying mechanisms of response in both hepatic and immune compartments through both single-cell and spatial approaches. Our findings provide the mechanistic basis for clinical investigations of this RNAi therapeutic targeting β-catenin for HCC treatment as a novel treatment paradigm in the form of monotherapy and/or in combination with immunotherapy in human subjects belonging to the Wnt-β-catenin active HCC subclass.

## RESULTS

### RNAi-mediated β-catenin Inhibition Results in Potent CTNNB1 Knockdown in vitro and in vivo

**Figure 1.**
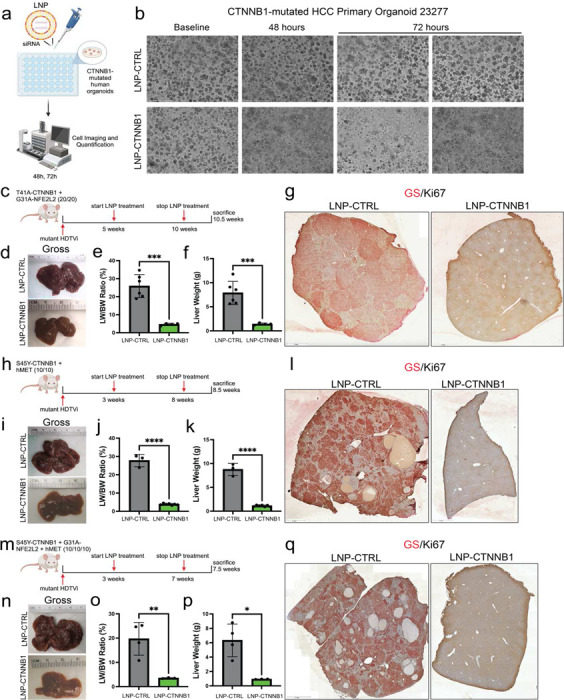
RNAi-mediated β-catenin inhibition is efficacious in multiple immunocompetent CTNNB1-mutated HCC mouse models in early-stage disease setting. (a)Schematic diagram demonstrating CTNNB1-mutated patient-derived HCC organoid treatment with LNP-CTRL or LNP-CTNNB1. (b)Brightfield microscopy images of CTNNB1-mutated patient-derived HCC organoids treated with LNP-CTRL or LNP-CTNNB1 at baseline, 48-hours, and 72-hours. Scale bar indicates magnification. (c) LNP treatment scheme in β-catenin-Nrf2 (β-N) model. Mice received once weekly intravenous (I.V.) injections at 1mg/kg dosage starting at 5-weeks post-hydrodynamic tail vein injection (HDTVi). (d)Representative gross liver images of LNP-CTRL and LNP-CTNNB1 treated β-N animals at 10.5-week timepoint. (e) Liver weight/body weight (LW/BW) ratio comparing LNP-CTRL (n=6) and LNP-CTNNB1 (n=4) treated β-N animals at 10.5-week timepoint. ***p<0.001 calculated by two-tailed Student’s t-test. (f) Liver weights comparing LNP-CTRL (n=6) and LNP-CTNNB1 (n=4) treated β-N animals at 10.5-week timepoint. ***p<0.001 calculated by two-tailed Student’s t-test. (g)Representative tiled images of immunohistochemistry (IHC) for glutamine synthetase (GS)/Ki67 co-stain comparing LNP-CTRL and LNP-CTNNB1 treated β-N animals at 10.5-week timepoint. Red stain is GS and nuclear brown stain is Ki67. Scale bar indicates magnification. (h)LNP treatment scheme in β-catenin-hMet (β-M) model. Mice received once weekly I.V. injections at 1mg/kg dosage starting at 3-weeks post-HDTVi. (i) Representative gross liver images of LNP-CTRL and LNP-CTNNB1 treated β-M animals at 8.5-week timepoint. (j) LW/BW ratio comparing LNP-CTRL (n=3) and LNP-CTNNB1 (n=7) treated β-M animals at 8.5-week timepoint. ****p<0.0001 calculated by two-tailed Student’s t-test. (k)Liver weights comparing LNP-CTRL (n=3) and LNP-CTNNB1 (n=7) treated β-M animals at 8.5-week timepoint. ****p<0.0001 calculated by two-tailed Student’s t-test. (l) Representative tiled images of IHC for GS/Ki67 co-stain comparing LNP-CTRL and LNP-CTNNB1 treated β-M animals at 8.5-week timepoint. Scale bar indicates magnification. (m) LNP treatment scheme in β-catenin-Nrf2-hMet (β-N-M) model. Mice received once weekly I.V. injections at 1mg/kg dosage starting at 3-weeks post-HDTVi. (n)Representative gross liver images of LNP-CTRL and LNP-CTNNB1 treated β-N-M animals at 7.5-week timepoint. (o)LW/BW ratio comparing LNP-CTRL (n=4) and LNP-CTNNB1 (n=3) treated β-N-M animals at 7.5-week timepoint. **p<0.01 calculated by two-tailed Student’s t-test. (p)Liver weights comparing LNP-CTRL (n=4) and LNP-CTNNB1 (n=3) treated β-N-M animals at 7.5-week timepoint. *p<0.05 calculated by two-tailed Student’s t-test. (q)Representative tiled images of IHC for GS/Ki67 co-stain comparing LNP-CTRL and LNP-CTNNB1 treated β-N-M animals at 7.5-week timepoint. Scale bar indicates magnification.

To study the effects of RNAi-mediated inhibition in β-catenin-mutated HCC, we utilized a novel siRNA that targets the *CTNNB1* gene, with both mouse and human specificity, encapsulated in a lipid nanoparticle (referred hereafter as LNP-CTNNB1). We first assessed whether LNP-CTNNB1 affected growth in a patient-derived HCC organoid (23277) with known mutation in *CTNNB1*.^25^ 72-hour treatment with LNP-CTNNB1 at 20nm concentration led to a notable decrease in both the number and size of the organoid compared to treatment with a LNP-CTRL (Figure 1a–b). Thus, LNP-CTNNB1 demonstrates efficacy in mutant-*CTNNB1* human HCC organoid cultures.

Next, to assess its pharmacodynamic effects, we first delivered LNP-CTNNB1 via tail vein intravenous (I.V.) injection to mouse livers which were transfected with human S45Y-mutant-*CTNNB1* gene (S45Y-*hCTNNB1* mice) via sleeping beauty hydrodynamic tail vein injection (SB-HDTVi) system. We have previously reported that mouse hepatocytes overexpressing mutant-β-catenin alone via SB-HDTVi method do not develop HCC,^26^ but require a secondary driver, such as hMet, Kras, or mutant-Nrf2 to induce HCC.^20,26,27^ After 4 treatments at 3mg/kg dosing in S45Y-*hCTNNB1* mice (Figure S1a), we observed an appreciable decrease in liver weight to body weight ratio (LW/BW ratio), which is consistent with the role of β-catenin in regulating liver growth and size (Figure S1b–d).^28,29^ Additionally, expression of two well-known β-catenin target genes via immunohistochemistry (IHC), GS and Cyclin D1 (CCND1), was absent throughout the liver lobule, indicating high *mCTNNB1* gene knockdown (Figure S1e–f). Moreover, Myc-tag (present on the S45Y-*hCTNNB1* plasmid) positive cells were absent throughout the liver parenchyma in LNP-CTNNB1 treated mice compared to islands of Myc-tag positive cells in LNP-CTRL mice, indicating high *hCTNNB1* gene knockdown (Figure S1e–f). Thus, LNP-CTNNB1 targets both endogenous mouse and mutant human *CTNNB1* with high potency and specificity *in vivo*.

Before testing efficacy of siRNA-mediated *CTNNB1* knockdown, we assessed whether there were any adjuvant effects of the LNP itself on the tumor immune microenvironment (TIME). We treated mice with either PBS, LNP-CTRL, or LNP-CTNNB1 utilizing a similar LNP frequency and dosage scheme as in Figure S1a, yet applied this to our T41A-mutant-β-catenin-Nrf2 (β-N) model (Figure S1g), which we have previously shown to represent 9–12% of all human HCC.^27^ Following treatment, we observed a decrease in liver weights and LW/BW ratio in LNP-CTNNB1 treated mice (Figure S1h–i), yet no appreciable difference in liver serum biochemistries (Figure S1j). Next, we performed bulk RNA-sequencing on all 3 treatment groups, and observed that PBS and LNP-CTRL treated animals are transcriptionally very similar, yet unique to the LNP-CTNNB1 treated animals (Figure S1k). Additionally, gene set enrichment analysis using gene ontology pathways demonstrated that the immune phenotype is similar between PBS and LNP-CTRL treated mice, suggesting the LNPs do not alter the immune excluded phenotype observed in CTNNB1-mutated HCC (Figure S1l).

### RNAi-mediated β-catenin Inhibition Impairs Tumor Growth in Multiple Immunocompetent CTNNB1-mutated and non-CTNNB1-mutated HCC Mouse Models with Durable Response in Early-stage Disease Setting

We next assessed the *in vivo* efficacy of LNP-CTNNB1 in *CTNNB1*-mutated and non-mutated HCC models. We first performed a dose titration study to determine lowest dose efficacy in our β-N model. We administered once weekly I.V. injections over 6 weeks of LNP-CTNNB1 starting at 5-weeks post-HDTVi, when microscopic tumor foci are established, at 3mg/kg, 1mg/kg, 0.3mg/kg, 0.1mg/kg, and 0.03mg/kg dosages (Figure S2a). There were significant tumor burden reductions across a wide LNP-CTNNB1 dose range (3mg/kg, 1mg/kg, 0.3mg/kg, and 0.1mg/kg), as evident by gross visualization and reduced LW/BW ratio (Figure S2b–f, Figure 1c–g). However, at 3mg/kg dosage, following the 4^th^ dose, we observed mortality in one of four mice, which was likely due to the high LNP dose and frequency. Additionally, the 0.3mg/kg, 0.1mg/kg, and 0.03mg/kg LNP-CTNNB1 dosages resulted in partial responses, with remnant microscopic tumor foci observed in 0.3mg/kg and 0.1mg/kg treated animals (Figure S2e) and macroscopic tumor nodules present in animals treated with 0.03mg/kg (Figure S2b, e). However, significant tumor responses were observed at the 1mg/kg dosage in LNP-CTNNB1 treated mice as noted via H&E, IHC for Myc-tag and GS/Ki67, and magnetic resonance imaging (MRI) (Figure 1g; Figure S3a–d). Thus, following this dose titration study in the β-N model, we determined that the 1mg/kg LNP-CTNNB1 dosage had profound *in vivo* efficacy for treatment of β-catenin-mutated HCC preclinical models without observable adverse effects.

To extrapolate our findings to additional β-catenin-mutated HCC preclinical models that we have previously reported, we next tested LNP-CTNNB1 in the more aggressive S45Y-mutant-β-catenin-Met (β-M) model, which represents 11% of human HCC.^26^ Here, we started treatment at 3-weeks post-HDTVi, a timepoint when microscopic tumor foci are established. Remarkably, following continued once weekly I.V. administration at 1mg/kg dosage over 6 weeks, there was a decrease in gross tumor burden (Figure 1h–l), and also a significant tumor response observed via H&E, Myc-tag, and GS/Ki67 IHC following LNP-CTNNB1 treatment (Figure 1l; Figure S3e–f). Moreover, starting at 3-weeks post-HDTVi, we tested LNP-CTNNB1 at the 1mg/kg dosage in a third *CTNNB1*-mutated model, the S45Y-mutant-β-catenin-Nrf2-Met (β-N-M) model, which represents ~5% of human HCC, independent of β-N and β-M models.^30^ Following a similar treatment protocol to the β-M model, we again observed significant tumor responses (Figure 1m–q; Figure S3g–h), similar to the results obtained in the “two-hit” models (β-N and β-M).

Lastly, we wanted to assess response to LNP-CTNNB1 in models that were not *CTNNB1*-mutated due to the general mitogenic function of Wnt-β-catenin signaling pathway in the liver.^31^ β-Catenin suppression by LNP-CTNNB1 in the Nrf2-hMet (N-M) model led to a decrease in LW/BW and in macroscopic disease (Figure S4a–d), yet there was persistence of microscopic nodules, which depicted inferiority in response when compared to mutant-β-catenin-driven tumors (Figure S4e). This decrease in tumor burden was observed despite HCC nodules in this model not homogenously positive for the bonafide Wnt target GS. We have also previously reported that c-Met/sgAxin1 tumors require intact β-catenin to initiate tumorigenesis.^32^ We also tested dependence on β-catenin in another independent non-*CTNNB1*-mutated HCC model using genetic approach (Figure S4h). β-Catenin deletion in SB-HDTVi induced Akt-NRas HCC in β-catenin floxed mice through simultaneous administration of pCMV-cre or control led to a significant improvement in overall survival and less tumor burden in pCMV-Cre compared to control, although tumors still persisted (Figure S4i). Thus, overall, we observed that β-catenin inhibition alone for *CTNNB1*-mutated HCC is most effective in early-stage disease setting as evident through significant tumor responses in multiple models of *CTNNB1*-mutated HCC, and as partial responses in β-catenin non-mutated HCC models.

Next, we assessed the long-term durability of the significant tumor responses observed in both the β-N and β-M models with LNP-CTNNB1 treatment at 1mg/kg dosage initiated at an early-stage disease treatment setting. Following the same treatment protocol in β-N (Figure 1c) and β-M (Figure 1h) models, we then withdrew LNP-CTNNB1. In the β-N model, treatment was ceased at 10 weeks, yet by ~22.5 weeks post-LNP-CTNNB1 treatment, gross tumor burden became equivalent to the tumor burden observed in mice with LNP-CTRL treatment at ~10.5 weeks which had been lethal in β-N mice (Figure S5a–b). Thus, with LNP-CTNNB1 treatment in β-N model, overall survival was significantly extended by ~12 weeks (*p*<0.001) (Figure S5c). The nodules that re-appeared at the ~22.5-week timepoint were positive for both GS and Nqo1 (Nrf2-target) (Figure S5d). In the β-M model, treatment was ceased at 8 weeks, yet by ~16.5 weeks post-LNP-CTNNB1 treatment, gross tumor burden was equivalent to that observed with LNP-CTRL treatment at the ~7.5 weeks which had been lethal in β-M mice (Figure S5e–f). Thus, LNP-CTNNB1 treatment in the β-M model extended overall survival by ~9 weeks (*p*<0.001) (Figure S5g). The nodules that reappeared at ~16.5-week timepoint in β-M model were positive for GS and V5-tag (present on hMet plasmid) (Figure S5h). Overall, LNP-CTNNB1 treatment as monotherapy more than doubled the survival of mice in both HCC models although tumors recurred after treatment cessation. These recurring tumors appear to be mutant-β-catenin-driven and not due to appearance of *de novo* resistant clones.

### Earliest Biological Response to RNAi-mediated β-catenin Inhibition Observed at 3-days Following Initial LNP-CTNNB1 Treatment

**Figure 2.**
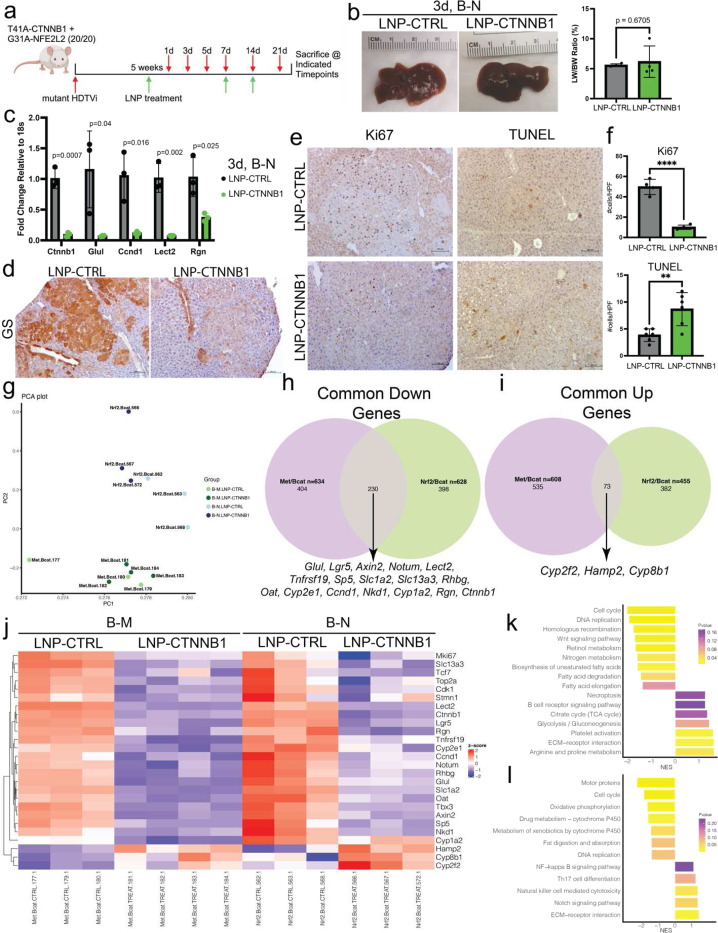
Earliest biological response to RNAi-mediated β-catenin inhibition observed 3-days after LNP treatment. (a)LNP treatment scheme in β-catenin-Nrf2 (β-N) model. Mice received 1^st^ LNP treatment at 1mg/kg dosage at 5-weeks post-hydrodynamic tail vein injection (HDTVi) via intravenous (I.V.) injection and sacrificed at 1-day, 3-days, 5-days, and 7-days post-LNP treatment. Then, 2^nd^ LNP treatment and sacrificed at 7-days later (14-days post 1^st^ LNP treatment). Then, 3^rd^ LNP treatment and sacrificed at 7-days later (21-days post 1^st^ LNP treatment). (b)(Left) Representative gross liver images of LNP-CTRL and LNP-CTNNB1 treated β-N animals at 3-days post 1^st^ LNP treatment. (Right) Liver weight/body weight (LW/BW) ratio comparing LNP-CTRL (n=4) and LNP-CTNNB1 (n=4) β-N treated animals 3-days post 1^st^ LNP treatment. *p*=0.6705 calculated by two-tailed Student’s t-test. (c)RNA expression levels of *Ctnnb1* and β-catenin target genes (*Glul, Ccnd1, Lect2, Rgn*) between LNP-CTRL (n=3) and LNP-CTNNB1 (n=3) β-N treated animals assessed by qPCR. *p<0.05 calculated by two-tailed Student’s t-test. Each data point is a biological replicate average of two technical replicates. (d)Representative immunohistochemistry (IHC) images for glutamine synthetase (GS) comparing LNP-CTRL and LNP-CTNNB1 treated β-N animals 3-days post 1^st^ LNP treatment. Scale bar is 100 m. (e)(Left) Representative IHC images for Ki67 and TUNEL comparing LNP-CTRL and LNP-CTNNB1 treated animals 3-days post 1^st^ LNP treatment. Scale bar indictaes 100 m. (f) Quantification of number of positive cells across multiple high-power fields (HPF) for Ki67 and TUNEL staining between LNP-CTRL and LNP-CTNNB1 treated β-N animals 3-days post 1^st^ LNP treatment. **p<0.01 calculated by two-tailed Student’s t-test. ****p<0.0001 calculated by two-tailed Student’s t-test. (g)Principal component analysis of bulk RNA-sequencing transcriptomic profiles of β-N and β-catenin-hMet (β-M) model each treated with LNP-CTRL or LNP-CTNNB1 and harvested 3-days post LNP treatment, using all genes (n=3–4 per condition and model). (h)Venn diagram highlighting number of common downregulated differentially expressed genes (DEGs) (n=230) between β-N and β-M models treated with LNP-CTRL or LNP-CTNNB1 3-days post LNP treatment. DEGs defined by FDR=0.05 and fold change > 1.5. (i) Venn diagram highlighting number of common upregulated DEGs (n=73) between β-N and β-M models treated with LNP-CTRL or LNP-CTNNB1 3-days post LNP treatment. DEGs defined by FDR=0.05 and fold change > 1.5. (j) Heatmap of selected common downregulated and upregulated genes demonstrating normalized z-score expression value in each model with each LNP treatment condition from (h) and (i). (k)Gene set enrichment analysis (GSEA) of Kyoto Encyclopedia of Genes and Genomes (KEGG) pathways based on differentially expressed genes (DEGs) in β-N model comparing LNP-CTRL and LNP-CTNNB1 treated animals. Pathways results demonstrate downregulating of cell cycle and Wnt signaling pathways and upregulation of immune and metabolic pathways. NES, normalized enrichment score. (l) GSEA of KEGG pathways based on DEGs in β-M model comparing LNP-CTRL and LNP-CTNNB1 treated animals. Pathways results demonstrate downregulating of cell cycle and drug metabolism pathways and upregulation of immune pathways. NES, normalized enrichment score.

Given the robust tumor responses following LNP-CTNNB1 treatment, we proceeded to investigate the earliest biological response observed following β-catenin knockdown within the tumor cells. In the β-N model, we followed mice over a 3-week treatment course (LNP-CTNNB1 injected weekly x 3) and sacrificed mice at 1-, 3-, 5-, 7-, 14-, and 21-days post the first treatment (Figure 2a). Over this 21-day treatment time course, the visible tumor foci or LW/BW ratio progressively trended lower in the LNP-CTNNB1 group although differences were insignificant (except day 5) when compared to time-matched LNP-CTRL group (Figure S6a; Figure 2b). However, at 3-days post a single LNP-CTNNB1 dose, RNA expression of *Ctnnb1*, along with Wnt target genes, *Glul*, *Ccnd1, Lect2*, and *Rgn* were significantly decreased in LNP-CTNNB1 mice compared to LNP-CTRL mice (Figure 2c). Additionally, GS protein expression visualized via IHC was decreased within tumor nodules, but retained in hepatocytes around central veins, at this 3-day timepoint, and by 14-days GS expression was absent in central vein hepatocytes in the LNP-CTNNB1 treated animals (Figure 2d; Figure S6b). Ki67 and TUNEL IHC also demonstrated significantly decreased tumor cell proliferation and increased cell death, respectively, at the 3-day timepoint, which was not observed at the 1-day timepoint (Figure 2e–f; Figure S6c–d). Given these results, we also administered a single treatment to β-M animals and sacrificed mice at 3-days post-treatment (Figure S7a). While there was no significant difference in gross tumor burden (Figure S7b), a single dose of LNP-CTNNB1 significantly decreased LW/BW ratio (Figure S7c–e), decreased intra-tumoral GS expression but retained V5-tag expression (Figure S7f–g). Also, there were significantly less intra-tumoral Ki67-positive cells and significantly more TUNEL-positive cells (*p*<0.01) (Figure S7h–i). Thus, the earliest evident biological response following RNAi-mediated β-catenin inhibition in both models occured at 3-days post-LNP treatment.

To understand the transcriptional consequences of β-catenin knockdown in HCC, we performed RNA-sequencing (RNA-seq) on both the β-N and β-M models treated with either LNP-CTRL or LNP-CTNNB1 at the 3-day timepoint. Each model clustered distinctly with LNP-CTNNB1 groups for each model clustering independently from the LNP-CTRL groups as shown via PCA analysis (Figure 2g). Differential gene expression analysis comparing LNP-CTRL vs LNP-CTNNB1 demonstrated 455 upregulated and 628 downregulated genes in the β-N model, and 608 upregulated and 634 downregulated genes in the β-M model, with 230 common downregulated and 73 common upregulated genes (Figure 2h–i). Common downregulated genes included Wnt/β-catenin target genes and pericentral marker genes (e.g., *Glul, Axin2, Lgr5, Notum, Lect2, Ccnd1, Cyp2e1, Cyp1a2*, and *Oat*), and common upregulated genes were midzonal and periportal marker genes (e.g., *Hamp2, Cyp8b1*, and *Cyp2f2*) (Figure 2j). From both models, Kyoto Encyclopedia of Genes and Genomes (KEGG) pathway analysis demonstrated positive enrichment of metabolic pathways, cell death pathways, immune activation pathways, NFβB signaling, and extracellular matrix signaling, along with negative enrichment of cell cycle, Wnt signaling pathways, fatty acid metabolism, retinol metabolism, and cytochrome P450 metabolic pathways (Figure 2k–l). Thus, we inferred β-catenin mutations in HCC confer most profound effects on tumor cell growth/proliferation, metabolism, and the tumor microenvironment.

### Integrated Single-Cell Analyses Reveal De Novo Formation of Reprogrammed Hepatocytes Within Remnant Tumor Nodules

To further interrogate tumor cell intrinsic biological effects that occurred at the 3-day timepoint, we administered LNP-CTRL or LNP-CTNNB1 at 5-weeks post-HDTVi to β-N model mice and performed single-cell RNA-sequencing (scRNA-seq) analysis on a hepatocyte-enriched single-cell population following whole liver perfusion. In total, 94,650 single cells were sequenced with 26,851 in the LNP-CTRL group and 67,799 in the LNP-CTNNB1 group. Unbiased clustering on the integrated dataset resulted in 10 unique cell populations (Figure S8a), annotated as a) Dying/injured hepatocytes, b), Hepatic stellate cells, c) Kupffer cells, d) Erythroid cells, e) Endothelial cells, f) Low-quality hepatocytes, g) Reprogrammed hepatocytes (expressing both zone 1 & 2 markers Ar*g1, Ass1, Pck1, Hal, Hamp2*, with Nrf2 tumor targets *Prdx2, Prdx5, Gstm1, Gpx1*), h) Zone 1 CTNNB1 WT (GS-negative) hepatocytes, i) Zone 1/2 CTNNB1 MUT (GS+) hepatocytes, and j) Zone 3 CTNNB1 WT & MUT (GS+) hepatocytes based on differential gene expression analysis per cluster (Figure S8b–c). KEGG pathway enrichment analysis comparing each hepatocyte cluster to all other clusters revealed that top pathways for Zone 3 CTNNB1 WT & MUT (GS+) hepatocytes were bile acid secretion, drug metabolism – cytochrome P450, and fatty acid metabolism, which are all known hallmarks of CTNNB1-mutated HCCs (Figure S8d).^33^ Zone 1/2 CTNNB1 MUT (GS+) hepatocytes and Zone 1 CTNNB1 WT (GS-negative) hepatocytes were interestingly enriched for arginine biosynthesis and amino acid biosynthesis KEGG pathways (Figure S8e–f), which are known metabolic hallmarks of zone 1 metabolism. This pathway analysis reveals the metabolic heterogeneity of tumor cells along the portal-central axis.

**Figure 3.**
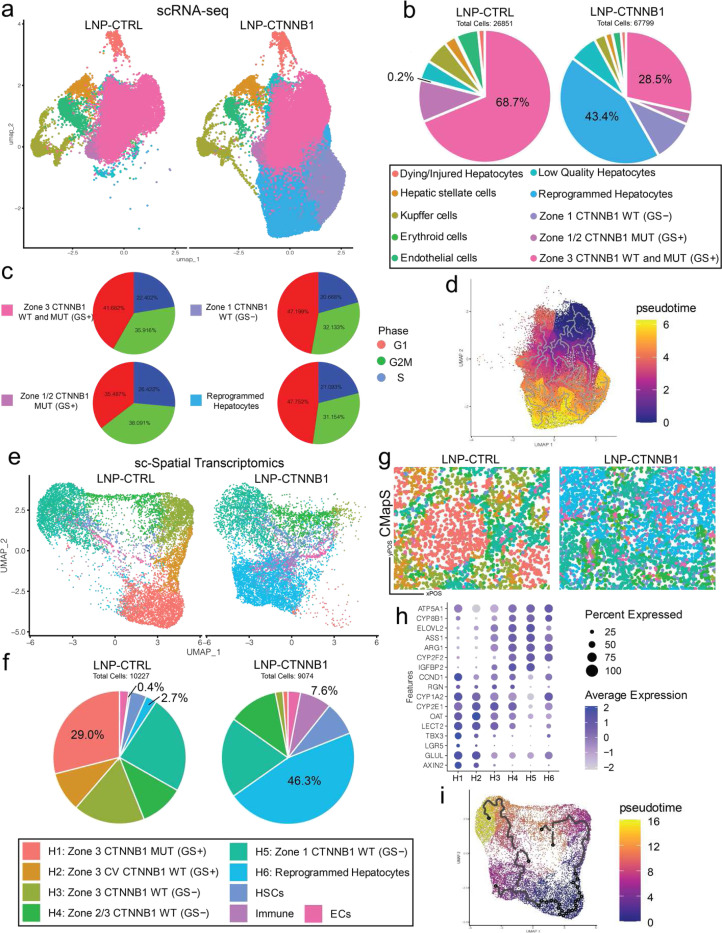
Integrated single-cell analyses reveal de novo formation of reprogrammed hepatocytes within remnant tumor nodules 3-days post LNP-CTNNB1 treatment. (a)Uniform manifold approximation and projection (UMAP) visualization of single-cell RNA-sequencing data following liver perfusion and enrichment of hepatocyte cell populations from LNP-CTRL and LNP-CTNNB1 treated β-catenin-Nrf2 (β-N) animals 3-days post LNP treatment. UMAP split by treatment condition with 94,650 cells total across both treatment conditions, n=2–3 pooled replicates each condition; LNP-CTRL (n=2) has 26,851 cells in the library; LNP-CTNNB1 (n=3) has 67,799 cells in the library after data integration. Labeled cell populations indicated by color. (b)Pie-chart of cell type proportions between LNP-CTRL and LNP-CTNNB1 treatment conditions from (a). Percentages of certain cell populations are indicated. Labeled cell populations indicated by color. (c) Cell cycle regression scoring for all cell population visualized via pie charts depicting cell cycle phase proportions in each of the hepatocyte cell clusters. Each pie piece represents a group of cells colored by whether the RNA expression fits cells belonging to G1 (red), G2/M (green), or S (blue) phases of the cell cycle. (d)Pseudotime trajectory analysis on UMAP plot subset to only hepatocyte specific cell populations using the Zone 3 CTNNB1 WT and MUT (GS+) cluster as the root. (e)UMAP visualization of single-cell spatial transcriptomic data via Molecular Cartogrpahy^™^ platform taken from frozen liver tissue sections of LNP-CTRL (n=1) and LNP-CTNNB1 (n=1) treated β-N animals 3-days post treatment. UMAP generated based on expression of 100 genes. UMAP split by treatment condition with 19,301 cells total across both treatment conditions (LNP-CTRL library has n=6 regions of interest (ROIs) with 10,227 cells total; LNP-CTNNB1 library has n=5 ROIs with 9,074 cells total). Labeled cell populations indicated by color. (f) Pie-chart of cell type proportions between LNP-CTRL and LNP-CTNNB1 treatment conditions from (f). Percentages of certain cell populations are indicated. Labeled cell populations indicated by color. (g)Cluster mapping to tissue section (CMapS) for LNP-CTRL and LNP-CTNNB1 virtual slides demonstrating visualization of certain cell populations by color from (f-g) on the actual tissue slide. (h)Dot plot visualization of various zonated marker gene expression (for all zones 1–3) for each hepatocyte cluster from (f-g). (i) Pseudotime trajectory analysis on Uniform manifold approximation and projection (UMAP) plot using the H1: Zone 3 CTNNB1 MUT (GS+) cluster as the root.

Cell-type proportion analysis comparing LNP-CTRL and LNP-CTNNB1 groups demonstrated less Zone 3 CTNNB1 WT & MUT (GS+) hepatocytes along with *de novo* appearance of reprogrammed hepatocytes following LNP-CTNNB1 treatment (Figure 3a–b). KEGG pathway analysis and gene set enrichment analysis on the reprogrammed hepatocytes demonstrated enrichment of pathways across all three liver lobule zones, including biosynthesis of cofactors (Zone 1), arginine biosynthesis (Zone 1), peroxisome (Zone 1), glutamate metabolism (Zone 3), glycolysis/TCA cycle (Zone 3), along with fatty acid metabolism, a pathway hallmark of CTNNB1-mutated hepatocellular cancers (Figure S9a–d). Cell cycle phase-specific gene expression analysis on hepatocyte clusters importantly demonstrated that tumor cells (both Zone 3 CTNNB1 WT & MUT [GS+] and Zone 1/2 CTNNB1 MUT [GS+] hepatocytes) were the most proliferative, while reprogrammed hepatocytes and Zone 1 CTNNB1 WT (GS-negative) hepatocytes were the least proliferative with proportionally fewer cells in G2M phase of the cell cycle (Figure 3c). In fact, reprogrammed hepatocytes and Zone 1 CTNNB1 WT were the two enriched hepatocyte populations following LNP-CTNNB1 treatment. Interestingly, Zone 1/2 CTNNB1 MUT [GS+] hepatocytes were the most proliferative tumor cell population, with the most cells in G2/M cell cycle phase (Figure S3c). We next performed pseudotime analysis on all the hepatocyte populations in this dataset to define cell states which demonstrated the intermediate cell state of these reprogrammed hepatocytes which occurred along the trajectory of Zone 3 CTNNB1 WT & MUT (GS+) hepatocytes to Zone 1 CTNNB1 WT (GS-negative) hepatocytes (Figure 3d). Thus, these reprogrammed hepatocytes are an intermediate cell phenotype, likely reflecting cancer cell differentiation to normal hepatocyte-like cells and contributing to the rapid cell turnover observed following LNP-CTNNB1 treatment.

Next, to confirm the spatial identity of these reprogrammed hepatocytes, we performed single-cell spatial transcriptomics using Molecular Cartography^™^ platform on tissue sections from the 3-day timepoint with LNP-CTRL or LNP-CTNNB1 treatment in the β-N model. The 100-gene panel consisted of markers specific for Wnt/β-catenin targets, metabolic zonation, and non-parenchymal cell types (see Methods). Following data pre-processing and automatic cell segmentation, in total, 19,301 single cells were sequenced from multiple regions of interest (ROIs) with 10,227 cells across 6 ROIs in LNP-CTRL group and 9,074 cells across 5 ROIs in LNP-CTNNB1 group. Unbiased clustering resulted in 9 unique cell populations, annotated as a) H1: Zone 3 CTNNB1 MUT (GS+), b) H2: Zone 3 Central Vein (CV) CTNNB1 WT (GS+), c) H3: Zone 3 CTNNB1 WT (GS-negative), d) H4: Zone 2–3 CTNNB1 WT (GS-negative), e) H5: Zone 1 CTNNB1 WT (GS-negative), f) H6: Reprogrammed hepatocytes, g) Hepatic stellate cells, h) Immune cells, and i) Endothelial cells, based on marker gene expression per cluster (Figure S10a–d). Clustering by treatment condition demonstrated similar enrichment of reprogrammed hepatocytes and loss of H1: Zone 3 CTNNB1 MUT (GS+) hepatocytes in LNP-CTNNB1 group (Figure 3e–f), similar to the scRNA-seq analysis (Figure 3a–b). Cluster Mapping to tissue Section (CMapS) confirmed the tumoral origin of the H6 cluster representing the reprogrammed hepatocytes (Figure 3g–h). In fact, spatial visualization and quantification of Wnt target genes revealed that β-catenin-mutated tumor cells are defined by expression of *Glul, Tbx3*, *Axin2, Lgr5, Lect2*, and *Ccnd1* (Figure S11a–b), along with their identity intimately linked to zone 3 metabolic genes (and processes), including *Cyp2e1*, *Cyp1a2*, and *Oat*, with exclusion of zone 1 metabolic genes (and processes), including *Cyp2f2, Ass1*, and *Arg1* (Figure S12a–b). However, with LNP-CTNNB1 treatment, tumor cells begin to express zone 1 markers, including *Cyp2f2, Arg1, and Ass1* (Figure S12a–b), while decreasing expression of zone 3 genes (e.g., *Cyp2e1, Cyp1a2*, and *Oat*). IHC validated these sc-Spatial transcriptomic findings and confirmed decreases in CYP2E1 and OAT, with increased expression of zone 1 markers ARG1 and CYP2F2, and zone 2 marker HAMP1/2 (Figure S12c). Additionally, pseudotime analysis on the sc-Spatial transcriptomic data confirmed the intermediary phenotype of the H6: reprogrammed hepatocytes (Figure 3i), as observed in the scRNA-seq data (Figure 3d). Lastly, for verification, cell cluster quantification was performed across each ROI within tumoral and non-tumoral regions (using *Glul* as tumoral landmark) (Figure S13a–b), which revealed a significant decrease in cell density of clusters with active β-catenin signaling, and significant increase in cell density of the H6: reprogrammed hepaotcytes cluster, which occurred mostly in tumoral regions (Figure S13c). Overall, this integrated single-cell analysis revealed that β-catenin-mutated tumor cells are exclusively zone 3 metabolic and respond to β-catenin suppression by turning off expression of these genes while differentiating towards zone 1/2 hepatocyte-like cells, thus reprogramming their metabolic machinery.

### Early β-catenin Suppression Induces an Innate Immune Response Characterized by Type I/II Interferon Network Signaling

**Figure 4.**
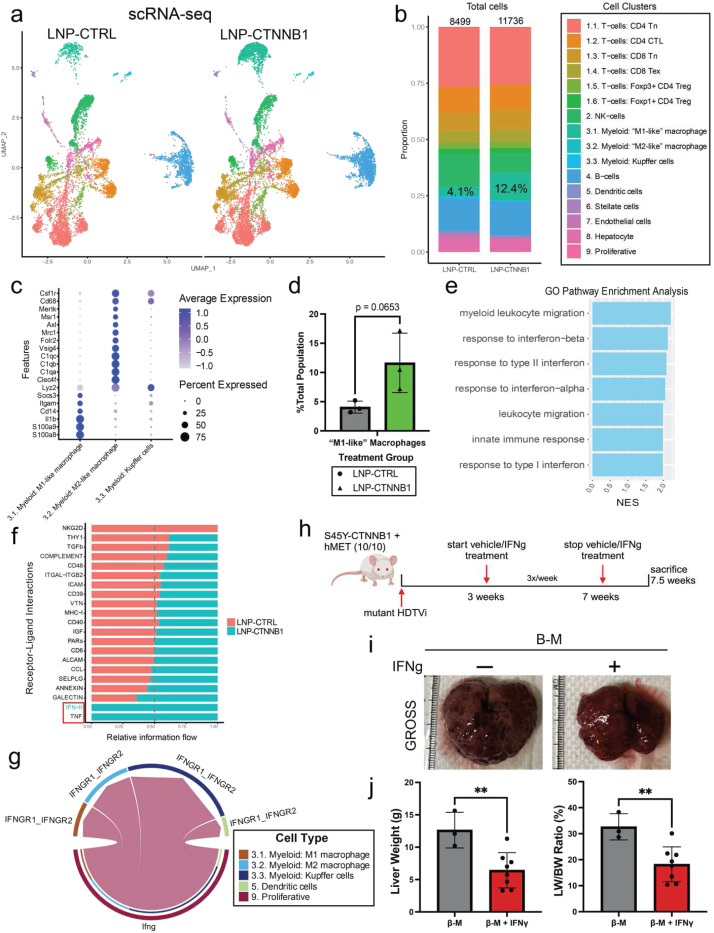
RNAi-mediated β-catenin inhibition induces pro-inflammatory myeloid cell intra-tumoral infiltration. (a)UMAP visualization of single-cell RNA-sequencing data following liver perfusion and enrichment of immune cell populations from LNP-CTRL and LNP-CTNNB1 treated β-N animals 3-days post LNP treatment. UMAP split by treatment condition with 20,235 cells total across both treatment conditions, n=3 biological replicates integrated for each condition; LNP-CTRL has 8,499 cells across n=3 replicates in the library; LNP-CTNNB1 has 11,736 cells across n=3 replicates in the library after data integration. Labeled cell populations indicated by color. (b)Stacked bar plot of cell type proportions between LNP-CTRL and LNP-CTNNB1 treatment conditions from (c). Labeled cell populations indicated by color. (c) Dot plot visualization of expression of canonical M1- and M2-macrophage phenotype markers in each of the different annotated cell populations. (d)Bar plot comparing average value of percent of total population of the M1-macrophage cell population between LNP-CTRL and LNP-CTNNB1 treatment. *p*=0.0653 calculated by two-tailed Student’s t-test. (e)Gene set enrichment analysis (GSEA) of Gene Ontology pathways based on genes differentially expressed in M1-macrophage population comparing LNP-CTRL and LNP-CTNNB1 treated animals. Pathways results demonstrate enrichment of pathways involved in interferon signaling response and innate immunity. NES, normalized enrichment score. (f) Stacked horizontal bar plot comparing relative information flow from CellChat between LNP-CTRL and LNP-CTNNB1 treated animals. Boxed pathways showing 100% information flow in LNP-CTNNB1 animals. IFN-II signaling highlighted in blue showing 100% enriched in LNP-CTNNB1 animals. (g)Cord diagram for IFN-II pathway in LNP-CTNNB1 treated animals demonstrating information flow from proliferative T cells to macrophage populations. No information flow in LNP-CTRL treated animals. (h)IFNβ treatment scheme in β-catenin-hMet (β-M) model. Mice received multi-weekly intra-peritoneal (I.P.) injections of IFNβ at 1×10^6^ IU/ml dosage or vehicle control starting at 3-weeks post-HDTVi. Mice were sacrificed at 7.5-weeks post-HDTVi. (i) Representative gross liver images of vehicle control and IFNβ treated β-M animals at 7.5-weeks post-HDTVi. (j) (Left) Liver weights comparing β-M animals treated with either vehicle control (n=3) and IFNβ (n=8) at 7.5-week timepoint. **p<0.01 calculated by two-tailed Student’s t-test. (Right) Liver weight/body weight (LW/BW) ratio comparing β-M animals treated with either vehicle control (n=3) and IFNβ (n=8) at 7.5-week timepoint. **p<0.01 calculated by two-tailed Student’s t-test.

CMapS also revealed more immune cells in the LNP-CTNNB1 group compared to the LNP-CTRL group (Figure 3g–h), which was also quantified (Figure S13c). To further investigate alterations in the immune landscape following LNP-CTNNB1 treatment in an unbiased manner, scRNA-seq was performed on an immune-enriched single-cell suspension from β-N treated animals. In total, 20,235 single cells were sequenced with 8,499 cells across 3 individual biological replicates in the LNP-CTRL group and 11,736 cells across 3 individual biological replicates in the LNP-CTNNB1 group. Unbiased clustering on the integrated dataset resulted in initially 21 unique clusters across the three biological replicates in the two treatment conditions (Figure S14a–b). To gain insights into the global immune cell changes, we combined and annotated the clusters as: a) T cells, b) B cells, c) NK cells, d) Hepatocytes, e) Myeloid, f) Proliferative, g) Dendritic cells, h) Endothelial cells, and i) Hepatic stellate cells, based on known marker gene expression for each of these cell types (Figure S14c–d). The majority cell populations that were ultimately sequenced were T cells, B cells, and Myeloid cells. We further subclustered and annotated these populations to better understand the T cell and myeloid cell functional states using marker genes previously described^34^ (Figure S15a–b; Figure 4a–c). The major differences observed following treatment were a 3-fold enrichment of “M1-like” pro-inflammatory macrophages in the LNP-CTNNB1 group (12.4%) compared to LNP-CTRL group (4.1%) (Figure 4b, d). At the 3-day time point following LNP-CTNNB1 treatment, we did not observe any significant differences in CD4 T cell populations in the β-N model from the immune-enriched scRNA-seq analysis (Figure S15c), or the sc-Spatial Transcriptomic analysis (Figure S15d–e). Additionally, in the the β-M model, IHC for CD4 did not reveal differences at the 3-day timepoint following LNP-CTNNB1 treatment (Figure S15e). Thus, innate immunity via myeloid cells, appear to be the predominant cell population which shifts 3-days post treatment (Figure 4d).

To investigate functional changes within the “M1-like” macrophage population, we performed differential gene expression comparing the “M1-like” macrophages from LNP-CTRL and LNP-CTNNB1 treatment. Gene ontology (GO) pathway analysis demonstrated enrichment of both response to type I/II interferon and interferon alpha/beta pathways following LNP-CTNNB1 treatment (Figure 4e). CellChat analysis, which determines pathway level changes based on gene expression of ligand-receptor interactions^35^, showed enrichment of IFN-II and TNF signaling in the “M1-like” macrophage population in the LNP-CTNNB1 treatment group (Figure 4f). Specifically, this analysis shows high probability of cell communication via Ifng from proliferative T cells with Ifngr1 and Ifngr2 on “M1-like” macrophages, and other macrophage cell populations solely in the LNP-CTNNB1 group (Figure 4g). Thus, increased type I/II interferons released from the immune compartment (likely from T cells and macrophages) following LNP-CTNNB1 treatment are engaging with macrophages in the TIME milieu, and in part contributing towards polarizing them towards a pro-inflammatory anti-tumor phenotype. To validate our findings that IFNβ is mediating an anti-tumor immune response following LNP-CTNNB1 treatment (Figure 4f–g), we treated β-M mice with IFNβ 3x weekly for 5 weeks, which led to a significant decrease in tumor burden compared to vehicle controls (Figure 4h–j). Thus, early β-catenin suppression induces recruitment of innate effector cells which mediate response to enhanced interferon network signaling driving an anti-tumor immune response.

### Mutated-β-catenin Represses a Module of Transcription Factors which Drives Immune Exclusion in CTNNB1-mutated HCC

**Figure 5.**
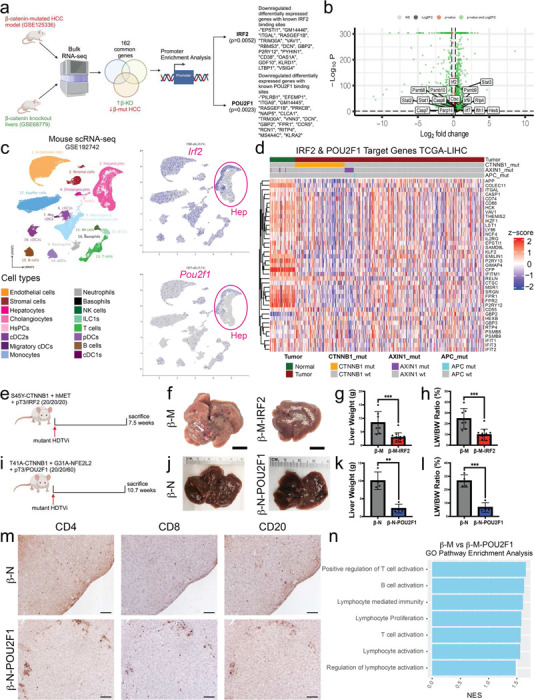
IRF2 and POU2F1 repression by mutated-β-catenin is a major tumor cell intrinsic mechanism of immune exclusion in CTNNB1-mutated HCC. (a)Schematic highlighting bioinformatic pipeline to compare whole transcriptome of β-catenin-mutated HCC (GSE125336) to β-catenin knockout livers (GSE68779) and focusing on 162 common genes downregulated in β-catenin-mutated HCC with absolute log fold change >2.0 and FDR=5%. Promoter enrichment analysis using JASPAR was performed on the downregulated genes with multiple transcription factors identified, including *Irf2* (p=0.0052) and *Pou2f1* (p=0.0023). (b)Volcano plot highlighting selected differentially expressed genes within the Zone 3 CTNNB1 WT and MUT (GS+) cell population comparing LNP-CTRL and LNP-CTNNB1 treatment from Figure 3a. Marker genes include *Irf2* and *Pou2f1* downstream target genes. (c) (Left) UMAP visualization of single-cell RNA-sequencing (scRNA-seq) data from GSE192742 (https://www.livercellatlas.org/index.php) of all mouse liver cells annotated by cell type. (Right) *Irf2* and *Pou2f1* expression by cell type on UMAP from (b) demonstrating hepatocytes express *Irf2* and *Pou2f1* in mouse liver. (d)Heatmap visualization of normalized expression of IRF2 and POU2F1 target genes in TCGA-LIHC patients (n=374; red) and adjacent normal (n=50; green). Data is stratified by CTNNB1-mutated patients (n=98; yellow), AXN1-mutated patients (n=18; purple), and APC-mutated patients (n=3; light blue). (e) β-catenin-hMet (β-M) animals were co-injected with either pT3 (empty vector) or IRF2 plasmid at time of hydrodynamic tail vein injection (HDTVi) and sacrificed at 7.5-weeks post-HDTVi. (f) Representative gross liver images of β-M animals co-injected with either pT3 (empty vector) or IRF2. Scale bar indicates 1 centimeter (cm). (g)Liver weights comparing β-M-pT3 (n=7) and β-M-IRF2 (n=12) animals at 7.5-week timepoint. ***p<0.001 calculated by two-tailed Student’s t-test. (h)Liver weight/body weight (LW/BW) ratio comparing β-M-pT3 (n=7) and β-M-IRF2 (n=12) animals at 7.5-week timepoint. ***p<0.001 calculated by two-tailed Student’s t-test. (i) β-catenin-Nrf2 (β-N) animals were co-injected with either pT3 (empty vector) or POU2F1 plasmid at time of hydrodynamic tail vein injection (HDTVi) and sacrificed at 10.7-weeks post-HDTVi. (j) Representative gross liver images of β-N animals co-injected with either pT3 (empty vector) or POU2F1. Scale bar indicates 1 centimeter (cm). (k)Liver weights comparing β-N-pT3 (n=4) and β-N-POU2F1 (n=4) animals at 10.7-week timepoint. **p<0.01 calculated by two-tailed Student’s t-test. (l) Liver weight/body weight (LW/BW) ratio comparing β-N-pT3 (n=4) and β-N-POU2F1 (n=4) animals at 10.7-week timepoint. ***p<0.001 calculated by two-tailed Student’s t-test. (m) Representative IHC images for CD4, CD8, and CD20 immune markers comparing β-N-pT3 and β-N-POU2F1 animals at 10.7-week timepoint. (n)Gene set enrichment analysis (GSEA) of Gene Ontology pathways based on genes differentially expressed in β-N-POU2F1 compared to β-N-pT3 animals. NES, normalized enrichment score.

Given the general amplified immune response early after LNP-CTNNB1 treatment, we next investigated potential tumor cell-intrinsic molecular mechanisms driving the immune excluded phenotype in β-catenin-mutated HCCs. We utilized bulk RNA-seq datasets which contained the transcriptome of multiple β-catenin-mutated HCC mouse models (GSE125336) and β-catenin knockout mouse livers (GSE68779) and performed transcription factor enrichment analysis on the 162 common genes which were downregulated in β-catenin-mutated HCC and upregulated in β-catenin knockout livers. We identified multiple transcription factors, including *Irf2* (*p*=0.0052) and *Pou2f1* (*p*=0.0023), as candidate transcription factors with known binding to he upregulated genes in β-catenin knockout livers (Figure 5a). To prioritize targets for potential therapeutic relevance, we further analyzed the scRNA-seq dataset (Figure 3a) and performed differential gene expression analysis on the Zone 3 CTNNB1 WT & MUT (GS+) hepatocyte cell population, and observed *Irf2* and *Pou2f1* target genes upregulated following LNP-CTNNB1 treatment (Figure 5b). To confirm whether tumor hepatocytes could be mediating IRF2 and POU2F1 downstream signaling to influence immune response, we investigated *Irf2* and *Pou2f1* expression in both human and mouse liver scRNA-seq datasets^36^ (GSE192742). We observed *Irf2/IRF2* and *Pou2f1/POU2F1* expression in hepatocyte cell populations in both mouse and human livers (Figure 5c; Figure S16a), suggesting that β-catenin-mediated IRF2 suppression may be a hepatocyte cell intrinsic process. Interestingly, expression of IRF2 and POU2F1 target genes in TCGA-LIHC cohort were notably downregulated in HCC patients with either *CTNNB1, AXIN1*, or *APC* mutations compared to those that did not have mutations known to confer β-catenin activation (Figure 5d). Thus, we hypothesized that mutated-β-catenin is a repressing a module of transcription factors (TFs) driving immune exclusion and limiting an anti-tumor immune response.

To validate that repression of IRF2, POU2F1, and other TFs are driving immune exclusion in β-catenin-mutated HCC, we first overexpressed either pT3 (empty vector) or *Irf2* (β-M-IRF2) in the β-M model (Figure 5e). We observed a significant decrease in overall tumor burden grossly at 7.5-weeks post-HDTVi and via decreased LW/BW ratio in *Irf2*-overexpression β-M model (Figure 5f–h). RNA-seq confirmed the overexpression of *Irf2* in the β-M-IRF2 mice at the 7.5-week timepoint where less tumor burden was evident (Figure S16b). Expectedly, given the known immunomodulatory roles of IRF2 and its involvement in type I/II interferon signaling^37^, we observed an increased presence of immune aggregates as evident by CD45 IHC (Figure S16c). This was validated with fluorescence-activated cell sorting (FACS) on isolated immune cells from β-M-pT3/β-M-IRF2 mouse HCC which demonstrated significant increases in total CD4+ cells with decreases in T regulatory populations in the β-M-IRF2 group (Figure S16d, S17a). Next, we overexpressed either pT3 (empty vector) or *Pou2f1* ( β-N-POU2F1) in the β-N model (Figure 5i). We also observed here a significant decrease in overall gross tumor burden at 10.7-weeks post-HDTVi in *Pou2f1*-overexpression β-N model (Figure 5j–l) and via histology (Figure S18a). These findings were also validated in the β-M model where significant reductions in tumor burden were observed at 7.7-weeks post-HDTVi in β-M-POU2F1 group (Figure S18b–e). Interestingly, IHC for CD4, CD8, and CD20 revelaed increased recruitment of T and B cells aggregating in the TIME in the β-N-POU2F1 group (Figure 5m). RNA-seq confirmed the overexpression of *Pou2f1* in the β-M-POU2F1 mice at the 7.7-week timepoint, along with decreased enrichment of our previously reported mutated-β-catenin gene signature (Figure S18f–h).^30^ Additionally, GO pathway analysis demonstrated enrichment of T and B cell activation and proliferation (Figure 5n). Lastly, given the less well characterized role of POU2F1 mediating an immune response, as compared to known functions of IRF2,^37,38^ we administered βCD3 to deplete CD3+ immune cells from β-M-POU2F1 mice (Figure S19a). Interestingly, at 8.3-weeks post-HDTVi, there was a significant increase in tumor burden in β-M-POU2F1 + βCD3 versus β-M-POU2F1 + IgG animals (Figure S19b–c), suggesting an immune-dependent role for POU2F1-mediated tumor regression in CTNNB1-mutated HCC. Overall, mutated-β-catenin represses IRF2, POU2F1, and likely other TFs, which limits transcription of key chemokines and cytokines important for priming recruitment of lymphocytes needed for an effective anti-tumor immunity and ICI response.

### RNAi-mediated β-catenin Inhibition Impairs Tumor Growth in Multiple Immunocompetent CTNNB1-mutated HCC Mouse Models in Late-stage Disease Setting with Response Associated with Restored Adaptive Immune Surveillance

**Figure 6.**
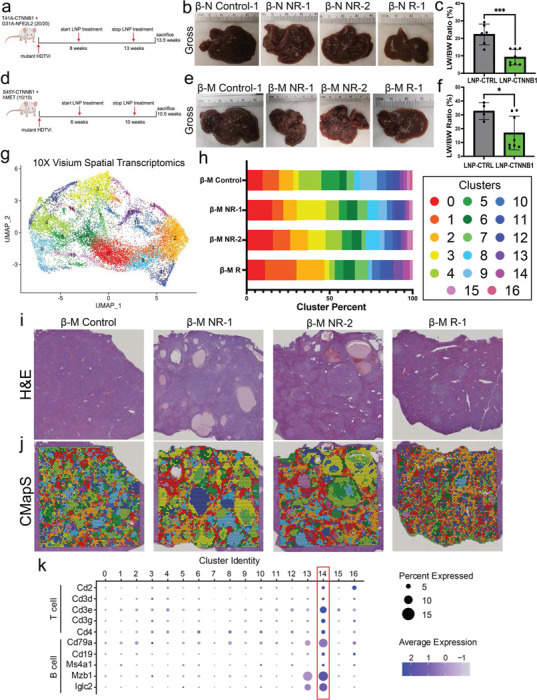
Response to RNAi-mediated β-catenin inhibition in CTNNB1-mutated mouse models in advanced stage disease setting is associated with overall T cell infiltration and activation. (a)LNP treatment scheme in β-catenin-Nrf2 (β-N) model for advanced-stage disease. Mice received once weekly intravenous (I.V.) injections at 1mg/kg dosage starting at 8-weeks post-hydrodynamic tail vein injection (HDTVi). (b)Representative gross liver images of LNP-CTNNB1 treated β-N animals at 13.5-week timepoint demonstrating non-responders (NR) and responders (R) compared to LNP-CTRL β-N animals when moribund at ~10.5-weeks. (c) Liver weight/body weight (LW/BW) ratio comparing LNP-CTRL (n=6) and LNP-CTNNB1 (n=8) treated β-N animals at 10.5-week and 13.5-week timepoint, respectively. ***p<0.001 calculated by two-tailed Student’s t-test. (d)LNP treatment scheme in β-catenin-hMet (β-M) model for advanced-stage disease. Mice received once weekly intravenous (I.V.) injections at 1mg/kg dosage starting at 6-weeks post-hydrodynamic tail vein injection (HDTVi). (e)Representative gross liver images of LNP-CTNNB1 treated β-M animals at 10.5-week timepoint demonstrating non-responders (NR) and responders (R) compared to LNP-CTRL β-M animals when moribund at ~8.5-weeks. (f) LW/BW ratio comparing LNP-CTRL (n=4) and LNP-CTNNB1 (n=8) treated β-M animals at 8.5-week and 10.5-week timepoint, respectively. *p<0.05 calculated by two-tailed Student’s t-test. (g)Uniform manifold approximation and projection (UMAP) visualization of gene expression profiles from 10X Visium spatial transcriptomics on the β-M model. Data integration was performed on 17,685 spots across 4 slides with 4,461 spots in β-M Control, 4,331 in β-M NR-1, 4,842 in β-M NR-2, and 4,051 in β-M R-1. Each color indicates one of the different 17 clusters. (h)Stacked bar chart of cluster proportions for each of the 4 slides from (g). Cluster number by color is indicated next to the graph. Each color indicates one of the different 17 clusters. (i) H&E slide from which the 10X Visium spatial transcriptomics was performed on for each of the 4 different conditions in the β-M model: β-M Control, β-M NR-1, β-M NR-2, and β-M R-1. (j) Cluster mapping to tissue section (CMapS) for each slide showing the cluster location on the H&E tissue section. (k)Dot plot showing expression by cluster for various T cell marker genes: *Cd2, Cd3d, Cd3e, Cd3g*, and *Cd4* and B cell marker genes: *Cd79a, Cd19, Ms4a1, Mzb1*, and *Iglc2* for the 10X Visium spatial transcriptomics data on the β-M model.

To assess the translatability of our findings to clinically relevant advanced-stage HCC, we next assessed the *in vivo* activity of LNP-CTNNB1 in late-stage disease CTNNB1-mutated HCC models, including both the β-N and β-M models. First, we assessed response to late-stage intervention in the β-N model where we administered once weekly I.V. LNP treatments starting at 8-weeks post-HDTVi to mimic clinically relevant advanced-stage disease (Figure 6a). Interestingly, after 6 cycles we observed a heterogenous response to LNP-CTNNB1 with 5/8 animals responding and 3/8 animals demonstrating poor response at 13.5-weeks post-HDTVi (Figure 6b–c; Figure S20a). Unsurprisingly, tumor foci in responder animals were less proliferative (evident via Ki67 IHC) and showed decreased expression of β-catenin (Myc-tag) and β-catenin targets, such as GS, via IHC (Figure S20b–c). Next, we studied response to LNP-CTNNB1 in the more aggressive β-M model with once weekly I.V. treatments starting at 6-weeks post-HDTVi to mimic clinically relevant advanced-stage disease (Figure 6d). Like the β-N model, we observed a heterogeneous response to LNP-CTNNB1 with 5/8 animals responding and 3/8 animals demonstrating no response at 10.5-weeks post-HDTVi (Figure 6e–f; Figure S21a). Similarly to the β-N model, we observed fewer tumor foci that were Myc-tag, GS/Ki67, and cyclin D1 positive in the responder animals (Figure S21b–d).

To investigate the mechanistic basis of the observed heterogeneous response, especially in the more aggressive β-M model, we employed the 10X Visium platform to perform unbiased spatial transcriptomics on an LNP-CTRL treated β-M HCC (“ β-M Control”), 2 LNP-CTNNB1 treated β-M HCC showing minimal/no response (“ β-M NR-1”; “ β-M NR-2”), and an LNP-CTNNB1 treated β-M HCC showing response (“ β-M R-1”). In total, we sequenced 17,685 spots across the 4 slides, with 4,461 spots in β-M Control, 4,331 in β-M NR-1, 4,842 in β-M NR-2, and 4,051 in β-M R-1. After integrating data from all slides, unbiased clustering revealed 17 clusters conserved across the different treatments (Figure 6g; Figure S22a–b). CMapS and cluster proportion analysis revealed increases in cluster 3 within tumor nodules in β-M NR animals, and increases in clusters 2, 13, and 14 in the β-M R animal (Figure 6h–j; Figure S22a–b). Given the lack of single cell specificity with the 10X Visium platform, we wanted to address pseudocell composition of these clusters, and performed differential gene expression per cluster compared to all other clusters (Figure S23a–q). To address mechanistic basis of response, we characterized clusters 2, 13, and 14 which were expanded in the β-M R animal. Cluster 2 expressed zone 1 and 2 metabolic genes, including *Cyp2f2, Pck1, Cps1*, and *Hamp* analogous to the reprogrammed tumor cell population observed in the early-stage LNP-CTNNB1 treatment setting (Figure S23c). Clusters 13 and 14 were enriched in lymphocyte markers (Figure S23n–o). Visualization of lymphocyte marker gene expression by cluster demonstrated enrichment of T and B cell genes in clusters 13 and 14 (Figure 6k; Figure S24a–b), with these 2 clusters enriched in the β-M R animal.

**Figure 7.**
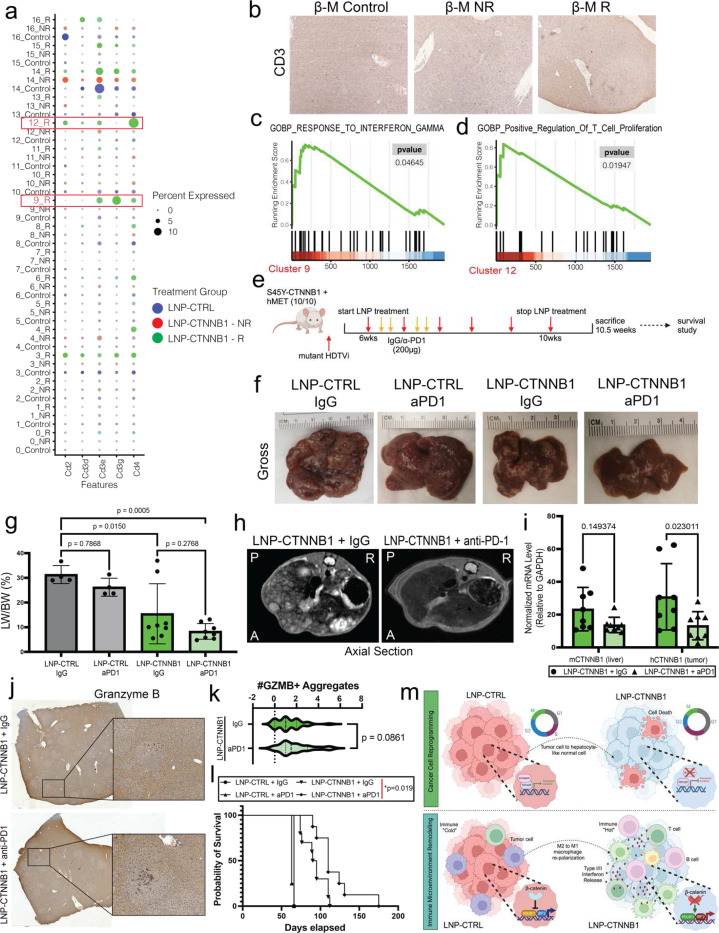
RNAi-mediated β-catenin inhibition synergizes with immunotherapy in β-M CTNNB1-mutated mouse model in advanced disease setting. (a)Dot plot T cell marker genes (Cd2, Cd3d, CD3e, Cd3g, Cd4) to visualize expression by cluster and by response (control [blue]; non-responder [NR; red], responder [R; green]) from the 10X Visium spatial transcriptomics data in figure 6d. (b)(Left) Representative immunohistochemistry (IHC) images from β-catenin-hMet (β-M) treated animals with LNP-CTRL or LNP-CTNNB1 (categorized by NR or R) at 10.5-week timepoint stained for CD3. 5X objective magnification for the images. (Right) Bar plot of quantification across multiple high-power fields (HPF) for CD3 in the tumor microenvironment for each of the different conditions. (c)Gene ontology (GO) gene set enrichment analysis (GSEA) running score plot for response to GOBP_Response_To_Interferon_Gamma in cluster comparing β-M Control (LNP-CTRL) to β-M R (LNP-CTNNB1; responder). (d)GO GSEA running score plot for GOBP_Positive_Regulation_Of_T_Cell_Proliferation in cluster 12 comparing LNP-CTRL to LNP-CTNNB1 R β-M animals. (e)LNP + IgG/β-PD1 treatment scheme in β-M model. Mice received weekly LNP injections at 1mg/kg dosage starting at 6-weeks post-hydrodynamic tail vein injection (HDTVi) with twice weekly injections of IgG/β-PD1 (200 g) for two weeks starting at 6-weeks post-HDTVi (3-days after LNP treatment). LNP-CTNNB1 treated mice were sacrificed at 10.5-weeks post-HDTVi or extended for survival analysis. (f) Representative gross liver images of LNP-CTNNB1 IgG/β-PD1 treated β-M animals at 10.5-week timepoint compared to LNP-CTRL IgG/β-PD1 when moribund. (g)Liver weight/body weight (LW/BW) ratio data comparing LNP-CTNNB1 IgG/β-PD1 (n=8/n=8) β-M treated animals at 10.5-week timepoint to LNP-CTRL IgG/β-PD1 (n=4/n=4) β-M treated animals when moribund. P-values calculated by one-way ANOVA with Tukey-HSD post-hoc comparison. (h)(Left) Magnetic resonance images (MRI) of LNP-CTNNB1 IgG/β-PD1 β-M treated animals at 10.5-week timepoint. (Right) Quantification represents area of 3-dimensional tumor volumes (defined by hyperintense foci) outlined comparing LNP-CTNNB1 IgG/β-PD1 β-M treated animals. (i) RNA expression levels of mCTNB1 and hCTNNB1 in LNP-CTNNB1 IgG/β-PD1 β-M treated animals (n=8 each group) assessed by qPCR. *p<0.05 calculated by two-tailed Student’s t-test. Each data point is a biological replicate average of two technical replicates. (j) Representative tiled immunohistochemistry (IHC) images from LNP-CTNNB1 IgG/β-PD1 β-M treated animals at 10.5-week timepoint stained for granzyme B (GZMB) to identify lymphoid aggregates with cytotoxic activity. Scale bar indicates magnification. (k)Violin plot for quantification of number of GZMB+ lymphoid aggregates in tumoral and peritumoral areas correlated with H&E lymphoid aggregate presence from LNP-CTNNB1 IgG/β-PD1 β-M treated animals at 10.5-week timepoint. P-value calculated by two-tailed Student’s t-test. (l) Kaplan-Meier survival curve of overall survival comparing β-M treated animals receiving LNP-CTRL IgG/β-PD1 (n=3/n=4) and LNP-CTNNB1 IgG/β-PD1 (n=10/n=8). Log-rank test was used to compare differences in mean overall survival time. *p=0.0188 comparing LNP-CTNNB1 + IgG to LNP-CTNNB1 + β-PD1. (m) Schematic diagram proposing two-part (i.e., cancer cell reprogramming and tumor immune microenvironment remodeling) working model for LNP-CTNNB1 treatment response mechanisms in β-catenin-mutated HCC preclinical models.

Given the role of T cells in promoting anti-tumor immunity, we examined expression of T cell marker genes *Cd2, Cd3d, Cd3e, Cd3g*, and *Cd4* by cluster and treatment response group, which revealed enrichment of *Cd3e*, *Cd3g*, and *Cd4* within β-M R animals in clusters 9 and 12 (Figure 7a), respectively, in which these tumor cell specific clusters decreased, compared to β-M Control and β-M NR animals (Figure 6h). This was also confirmed via IHC which demonstrated increased CD3+ cells throughout tumors and organized into lymphoid aggregates in β-M R animals (Figure 7b). GO GSEA demonstrated significant enrichment of response to IFNβ in cluster 9 and positive regulation of T cell proliferation in cluster 12 (Figure 7c–d; Figure S24c–d). To further discern the enhanced adaptive anti-tumor immune surveillance in β-M R animals, we performed spatially enhanced CellChat^35^ analysis to investigate ligand-receptor interactions between different clusters and within different treatment response groups. This analysis revealed enrichment of MHC-II signaling with antigen communication from most clusters to CD4+ cells in cluster 12 (tumor cluster) only in β-M R animals compared to both β-M Control and β-M NR animals (Figure S25a–d). Overall, β-M R animals demonstrate reinvigorated and persistent adaptive immune surveillance with active T and B cell infiltration, T cell proliferation, and engaged IFNβ signaling in intra-tumoral compartments, which likely was not sustained long-term in the NR phenotype in advanced disease setting.

### RNAi-mediated β-catenin Inhibition Synergizes with Immunotherapy in Advanced Disease Setting in CTNNB1-mutated HCC Mouse Model

We next investigated if administration of both LNP-CTNNB1 and ICI in late-stage HCC would synergize and promote long-term anti-tumor immunity. We posit that the NR phenotype during late-stage HCC LNP-CTNNB1 treatment reflected a lack of sustained active lymphocyte proliferation, infiltration, and response to IFNβ signaling in the intra-tumoral compartment. Following a similar scheme for advanced-stage disease LNP treatment in the β-M model, we added IgG or β-PD1 to the regimen 3-days after LNP dose, which was determined based on enhanced IFN signaling at this timepoint, and harvested mice by 10.5-week timepoint or when moribund to assess and compare response, and also performed a survival study to determine long-term anti-tumor immunity (Figure 7e). By the 10.5-week timepoint, LNP-CTRL mice were all moribund with β-PD1 alone not impacting tumor burden, yet the combination of LNP-CTNNB1 + β-PD1 resulted in enhanced efficacy with absence of any non-responders compared to LNP-CTNNB1 + IgG treated animals (Figure 7f–g). Additionally, MRI demonstrated less hyperintense foci in LNP-CTNNB1 treated mice receiving β-PD1 compared to IgG treatment (Figure 7h). Interestingly, *hCTNNB1* knockdown was enhanced in the LNP-CTNNB1 treated mice receiving β-PD1 compared to IgG treatment (*p*=0.02) suggesting an augmented response with β-PD1 (Figure 7i). To investigate potential mechanisms of LNP-CTNNB1 + β-PD1 synergy we performed IHC for granzyme B (GZMB) to address cytotoxic T cell activity and observed an overall increase in GZMB+ lymphoid aggregates within and surrounding remnant tumor nodules in LNP-CTNNB1 treated mice receiving β-PD1 compared to IgG treatment (*p*=0.08) (Figure 7j–k), suggesting improved anti-tumor immunity in mice receiving combination therapy. Concomitantly, mice receiving LNP-CTNNB1 + β-PD1 survived significantly longer than those receiving LNP-CTNNB1 + IgG (*p*=0.02) or either of the LNP-CTRL treatment groups (Figure 7l), suggesting synergy of β-catenin suppression with immunotherapy.

### TLS/LA are Enriched in Atezolizumab plus Bevacizumab Responders and CTNNB1-wild-type Patients in IMbrave150 Trial and Associated with Survival

**Figure 8:**
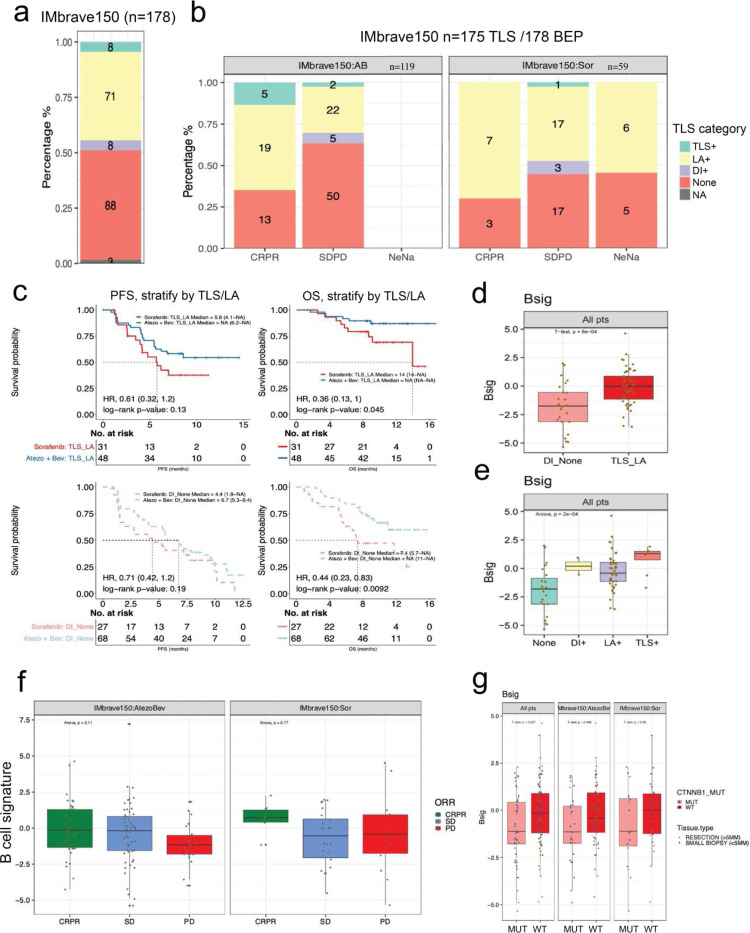
Lymphoid aggregates are prognostic in hepatocellular carcinoma and negatively correlated with CTNNB1 mutation. (a)Stacked bar graph depicting number of patients in IMbrave150 phase III trial having either TLS, LA, DI, None or NA from the total population of 178 patients. Ultimately, 175 HCC cases were in the biomarker evaluable cohort. (b)Stacked bar graph depicting number of patients in IMbrave150 phase III trial having either TLS, LA, DI, None or NA in each of the two treatment arms: atezolizumab + bevacizumab (n=119) versus sorafenib (n=59). (c) (Top Left) Kaplan-Meier survival curve for progression-free survival (PFS) comparing patients with TLS/LA in atezolizumab + bevacizumab versus sorafenib arms, demonstrating that TLS/LA presence trends towards improved PFS in atezolizumab + bevacizumab arm. (Bottom Left) Kaplan-Meier survival curve for PFS comparing patients with DI/None in atezolizumab + bevacizumab versus sorafenib arms, demonstrating DI/None is not prognostic. (Top Right) Kaplan-Meier survival curve for overall survival (OS) comparing patients with TLS/LA in atezolizumab + bevacizumab versus sorafenib arms, demonstrating that TLS/LA presence results in significantly improved OS in atezolizumab + bevacizumab arm. (Bottom Right) Kaplan-Meier survival curve for OS comparing patients with DI/None in atezolizumab + bevacizumab versus sorafenib arms, demonstrating that any immune cell presence results in significantly improved OS with atezolizumab + bevacizumab compared to sorafenib. Log-rank test was used to compare differences in survival outcomes. (d)Composite average expression of previously reported B cell signature5 stratified by whether patients in IMbrave150 phase III trial had TLS/LA or DI/None. Student t-test demonstrated significant increase of B cell signature in patients with TLS/LA compared to patients with DI/None (*p*<0.001). (e)Composite average expression of previously reported B cell signature^5^ stratified by whether patients in IMbrave150 phase III trial had TLS, LA, DI, or None. One-way ANOVA demonstrated significant increase of B cell signature in patients with TLS/LA compared to patients with DI/None (*p*<0.001). (f) Bar plot depicting B cell signature stratified by mRECIST response criteria (complete/partial [CR/PR]), stable disease (SD), and progressive disease (PD) in each of the treatment arms from IMbrave150 phase III trial. Increased B cell signature expression score was observed in both treatment arms, but more pronounced in atezolizumab + bevacizumab arm. (g)Bar plot depicting B cell signature stratified by CTNNB1 mutational status in all patients and within each of the two treatment arms from IMbrave150 phase III trial. Student t-test demonstrated significant increase of B cell signature in patients without CTNNB1 mutation compared to those with CTNNB1 mutation (p=0.027).

Given the restored adaptive immune surveillance and lymphoid aggregate (LA) presence upon β-catenin knockdown, we were interested whether there was a relationship between tertiary lymphoid structure (TLS)/LA, *CTNNB1* mutation, and ICI response from the IMbrave150 phase III clinical trial. In this trial of 178 HCC patients in the biomarker-evaluable population (BEP), 175 were scored by a clinical pathologist for presence of immune infiltration (TLS, LA, diffuse infiltrate [DI], and none) from hematoxylin & eosin (H&E) slides. Overall, majority of patients, irrespective of treatment arm, had LA (n=71/175), while fewer had TLS (n=8/175) or DI (n=8/175) (Figure 8a). Interestingly, among responders, those in the atezolizumab plus bevacizumab arm tended to be enriched for presence of TLS/LA, which was not observed in the sorafenib arm (Figure 8b). Additionally, patients with TLS/LA correlated with improved progression-free (PFS) and overall survival (OS), which was more pronounced in the atezolizumab plus bevacizumab arm (Figure 8c). Moreover, patients with TLS/LA had significantly increased expression of a previously reported B cell signature (Bsig), which was found to be correlated with TLS/LA presence in head and neck cancer,^39^ compared to patients with DI/None (Figure 8d–e). Increased Bsig expression was also observed in atezolizumab plus bevacizumab arm in patients with CR/PR and SD, while decreased Bsig expression was observed in those with PD (Figure 8f). Interestingly, Bsig was not associated with response in the sorafenib arm, indicating that TLS/LA recruitment may be primed with atezolizumab plus bevacizumab combination (Figure 8f). Lastly, we observed that *CTNNB1*-mutated patients had significantly lower Bsig expression compared to *CTNNB*-wild-type patients (Figure 8g). Thus, formation of TLS/LA may be restricted by mutated-β-catenin due to repression of various TFs in HCC affecting overall response to combination ICI.

## DISCUSSION

We report strong *in vitro* and *vivo* efficacy of a novel LNP-formulated siRNA targeting *CTNNB1* mRNA transcript for treatment of β-catenin-mutated HCC as monotherapy in early-stage disease or in combination with ICI at late-stage disease. We identified through unbiased scRNA-seq and spatial transcriptomic approaches a novel tumor-cell intrinsic role of β-catenin-mediated IRF2 and POU2F1 repression driving an immune excluded TIME and inert type I/II interferon responses in β-catenin-mutated HCC with *in vivo* validation. Additionally, we demonstrate upon β-catenin suppression, β-catenin-mutated tumor cells reprogram towards zone 1/2 hepatocyte-like cells, revealing the novel role of mutated-β-catenin in driving zone 3 (pericentral) tumor metabolism. Our work demonstrates that β-catenin is now targetable in murine HCC to overcome ICI resistance and supports the high impact development of clinical investigations utilizing LNP-CTNNB1 as a monotherapy or in combination with ICI to achieve therapeutic benefit in HCC patients with Wnt/β-catenin activation.

β-catenin is most active in the pericentral (zone 3) region in the hepatic lobule with hepatocytes in each of the three zones of the hepatic lobule expressing genes important for different metabolic functions, known as liver metabolic zonation.^33^ Given the localization of β-catenin to zone 3, it is no surprise that β-catenin-mutated tumors preferentially originate and clonally expand from hepatocytes residing within zone 3, and these tumors share unique metabolic addictions to processes canonically identified in zone 3. In fact, we have previously shown that CTNNB1-mutated HCC is addicted to glutamine synthesis,^40^ as part of β-catenin-GS-mTOR axis.^21^ Additionally, CTNNB1-mutated HCCs demonstrate addiction to xenobiotic metabolism through GSTM3.^41^ However, surprisingly, tumors with β-catenin oncogenic activation are not glycolytic (zone 3 metabolism), but are fatty acid oxidative (zone 1 metabolism) addicted.^42^ Here, we show that β-catenin-mutated tumors residing specifically in zone 3 are metabolically wired to perform canonical zone 3 metabolic processes with a focus on fatty acids as substrates, while β-catenin-mutated tumor cells in zone 1 are metabolically wired to perform canonical zone 1 metabolic processes with a focus on arginine metabolism and amino acid biosynthesis. We have also uniquely demonstrated that β-catenin-mutated tumor cells in zone 1 possess the highest proliferative capacity compared to those in zone 3, suggesting that despite β-catenin-mutated HCCs being well-differentiated, less proliferative tumors, in ectopic regions of absent Wnt signals or in presence of normal zone 1 signals, proliferation may be favored over metabolic homeostasis. Whether zone 1 β-catenin-mutated HCCs in current model are due to clonal expansion, evolution, or budding from zone 3 tumors to eventually establish in zone 1, or an artifact of plasmid transfection in rare hepatocytes in zone 1 requires further investigation. However, despite these tumor intrinsic pathways, the overall tumor biology and metabolism may also be regulated by local zonal environment and signals. Overall, we demonstrate that suppressing β-catenin in CTNNB1-mutated tumors reprograms zone 3 tumors towards a zone 1/2 metabolic phenotype as early as 3-days post LNP treatment, which contributes to the phenotypic differentiation and metabolic re-wiring, loss of tumor nodules, and normalization of hepatic parenchyma and liver mass. Such reprogramming may yield novel metabolic vulnerabilities to be exploited for additional therapies in the future.

Cancers with Wnt/β-catenin activation are considered non-T cell-inflamed across a variety of tumor types, including HCC, melanoma, esophageal, and others.^6,7,43,44^ This has been associated with resistance to ICIs, specifically of the anti-PD-1/anti-PD-L1 class of agents.^43^ Preclinical studies with genetic mouse models have revealed tumor-intrinsic roles of β-catenin regulating expression of transcription factor (TF) repressors (e.g., ATF3), which in turn modulate expression of crucial chemokine genes, including CCL4 and CCL5, involved in T cell priming and recruitment to the TIME.^7,44^ In HCC, many key chemokines are lowly expressed in CTNNB1-mutated patients, suggesting that potentially alternative mechanisms other than direct transcriptional repression may explain this phenomenon, given that β-catenin-TCF/LEF complex does not have binding sites at promoter regions for all these chemokines.^7^ In KRAS-mutated colorectal cancer, where ICI is also ineffective, expression of chemokines involved in IFN network signaling, such as CXCL3, were found to be mediated through KRAS-mediated interactions with IRF2.^37^ Here, we identified a novel tumor cell-intrinsic interaction of β-catenin/IRF2 where IRF2 (and IFN network signaling) is suppressed in β-catenin-mutated HCC. We demonstrate that β-catenin suppression directly increases IRF2 expression in β-catenin-mutated HCC models, with subsequent increases in interferon signaling molecules and antigen presentation machinery components. Additionally, we demonstrate that forced expression of IRF2 in β-catenin-mutated HCC model is sufficient to convert a non-T cell-inflamed to T cell-inflamed tumor. Given that our unbiased bioinformatic analysis identified other putative TFs, including POU2F1, whose function may be modulated in the context of β-catenin-mutated livers, we posit there exist an immune-regulatory module of TFs suppressed by mutated-β-catenin which modulates expression of key cytokines and chemokines involved in immune response, possibly in other tumor types as well. In fact, we and others have previously described the role of β-catenin in sequestering NF-βB, resulting in immune exclusion.^45–47^ Thus, pharmacologic targeting of β-catenin likely has clinical implications across a broad spectrum of tumor types to improve ICI clinical efficacy in part through modulation of key TFs involved in priming immune recruitment and engaging in global adaptive immune surveillance.

We have shown here that targeting β-catenin directly impacts both tumor cell intrinsic biology and simultaneously reprograms the TIME from non-T cell-inflamed to T cell-inflamed, with innate immune remodeling occurring as early as 3-days post LNP treatment. This innate immune remodeling coincided with first observed biological effect of β-catenin knockdown at 3-days. Biological effects due to siRNA knockdown are usually observed within hours *in vitro*,^48^ yet we observed a protracted time course in vivo, likely due to the systemic delivery method. Additionally, prior work has illustrated that adaptive immune surveillance begins to remodel at least 7–10 days following oncogene withdrawal, which explains the significant adaptive immune effects we observed studying late-stage response after 6 weeks of LNP treatment.^49^ However, the profound anti-tumor effects we observed here likely would not be so pronounced through targeting downstream effector molecules of the Wnt/β-catenin signaling pathway. Specifically, we and others have previously shown that genetic deletion or pharmacologic inhibition of downstream effectors of β-catenin-TCF/LEF interactions, such as cyclin D1 (encoded by CCND1),^50^ GS,^16^ mTORC1,^21^ TBX3,^17^ AXIN2^51^, or TNFRSF19^52^ either result in partial tumor responses or compensatory negative feedback loops leading to enhanced tumorigenesis. For example, it has been shown that hepatocarcinogenesis is not dependent on cyclin D1 as β-catenin-mutated tumors induced in Ccnd1-null background mice still develop through compensatory cyclin D2 expression.^50^ Additionally, conditionally deleting TBX3 or GS in mice with β-catenin-mutated HCC exacerbates tumorigenesis through YAP/TAZ inhibition or nitrogen metabolic rewiring, respectively.^16,17^ Moreover, our group has previously identified metabolic addiction to β-catenin-GS-mTOR axis in β-catenin-mutated HCC and evaluated mTOR inhibitor (e.g., rapamycin, everolimus) response in multiple preclinical models of β-catenin-mutated HCC. However, response to LNP-CTNNB1 results in more consistent, robust, and durable responses in preclinical models.^21,27^ Lastly, targeting solely TNFRSF19 will likely impact expression of chemokines involved in immune recruitment, yet there would be minimal impact on intrinsic tumor cell biology.^52^ Thus, targeting β-catenin directly is a holistic and rational strategy leading to durable anti-tumor immune responses through inhibiting multiple mechanisms hitting a truncal event, and impacting not only tumor-cell intrinsic biology, but also simultaneously remodeling the TIME architecture to promote long-lasting anti-tumor immunity.

Therapeutic targeting of Wnt/β-catenin oncogenic signaling has been pursued over the last two decades with no therapeutic agent ultimately resulting in translation to the clinic. First, given the ubiquitous role of β-catenin in many cell types, translation of many agents has been limited due to on-target, off-tumor effects.^43,53^ Small-molecule inhibitors which limit interactions between β-catenin and TCF/LEF or β-catenin and cAMP response element–binding protein (CREB)–binding protein (CBP), or repurposed drugs against Wnt activity have shown *in vitro* inhibitory effects, yet lack strong *in vivo* efficacy, likely due to alternative escape mechanisms.^9^ Alternative methods of Wnt/β-catenin inactivation have investigated porcupine (PORCN), tankyrase (TNKS), or Frizzled (FZD) receptor inhibitors, however, these are ineffective and far too upstream in the pathway for treating tumors with GOF CTNNB1 mutations due to subsequent independence of Wnt/FZD receptor binding.^9^ Thus, RNAi- or antisense-mediatred gene silencing approaches have proven to be an effective therapeutic approach to reduce CTNNB1 mRNA levels in tumors. Efficacy has previously been shown by our group and others across a variety of different tumor types.^20,22,23,54^ Our work here builds upon these previous findings and demonstrates that RNAi-mediated β-catenin inhibition via LNP for HCC results in minimal off-target effects with strong and durable on-target effects.

In summary, we propose a synergistic two-part working mechanism of response to RNAi-mediated β-catenin inhibition in preclinical CTNNB1-mutated HCC models (Figure 7m). First, early response to LNP-CTNNB1 treatment includes cessation of tumor cell proliferation and concomitant metabolic zonal reprogramming with zone 3 tumor cells converting to zone 1/2 hepatocytes. Second, cancer cell reprogramming simultaneously occurs with conversion of an immunologically cold to hot TIME in which macrophages repolarize from a M2-like to M1-like-phenotype in the tumor immune compartment and mediate potent anti-tumor immune responses. Simultaneously, IRF2 and POU2F1 re-engagement in the tumoral compartment, when β-catenin is suppressed, acts as a mediator of enhanced interferon network signaling and primes lymphocyte recruitment and infiltration, with all these tumor-intrinsic and TIME remodeling mechanisms ultimately driving synergy with β-PD1 in the advanced-stage disease setting. Based on our findings, RNAi-mediated inhibition of β-catenin may have the potential to provide anti-tumor effects as a monotherapy in early stage disease or in neoadjuvant setting in patients with Wnt-β-catenin active liver tumors. These proof-of-concept studies also support the clinical investigation of RNAi therapeutic approaches targeting β-catenin in combination with ICI in advanced-stage Wnt-β-catenin active-HCC patients.

## Supplementary Material

Supplement 1

## SIGNIFICANCE:

β-catenin is currently an “undruggable” target. Thus, utilizing a novel LNP-encapsulated siRNA targeting β-catenin, we demonstrate its efficacy for precision therapy in aggressive preclinical models, mechanisms underlying β-catenin-mediated immune escape, and synergy with ICI, paving a way forward for clinical trials.

## References

[1] Sung H, Ferlay J, Siegel RL, Global Cancer Statistics 2020: GLOBOCAN Estimates of Incidence and Mortality Worldwide for 36 Cancers in 185 Countries. CA Cancer J Clin. 2021;71(3):209–249. 33538338 10.3322/caac.21660

[2] Cheng AL, Qin S, Ikeda M, Updated efficacy and safety data from IMbrave150: Atezolizumab plus bevacizumab vs. sorafenib for unresectable hepatocellular carcinoma. J Hepatol. 2022;76(4):862–873. 34902530 10.1016/j.jhep.2021.11.030

[3] Sangro B, Chan SL, Kelley RK, Four-year overall survival update from the phase III HIMALAYA study of tremelimumab plus durvalumab in unresectable hepatocellular carcinoma. Ann Oncol. 2024. 10.1016/j.annonc.2024.02.005 38382875

[4] Rimassa L, Finn RS, Sangro B. Combination immunotherapy for hepatocellular carcinoma. J Hepatol. 2023;79(2):506–515. 36933770 10.1016/j.jhep.2023.03.003

[5] Sangro B, Sarobe P, Hervas-Stubbs S, Melero I. Advances in immunotherapy for hepatocellular carcinoma. Nat Rev Gastroenterol Hepatol. 2021;18(8):525–543. 33850328 10.1038/s41575-021-00438-0 PMC8042636

[6] Zhu AX, Abbas AR, de Galarreta MR, Molecular correlates of clinical response and resistance to atezolizumab in combination with bevacizumab in advanced hepatocellular carcinoma. Nat Med. 2022;28(8):1599–1611. 35739268 10.1038/s41591-022-01868-2

[7] Ruiz de Galarreta M, Bresnahan E, Molina-Sanchez P, beta-Catenin Activation Promotes Immune Escape and Resistance to Anti-PD-1 Therapy in Hepatocellular Carcinoma. Cancer Discov. 2019;9(8):1124–1141. 31186238 10.1158/2159-8290.CD-19-0074 PMC6677618

[8] Harding JJ, Nandakumar S, Armenia J, Prospective Genotyping of Hepatocellular Carcinoma: Clinical Implications of Next-Generation Sequencing for Matching Patients to Targeted and Immune Therapies. Clin Cancer Res. 2019;25(7):2116–2126. 30373752 10.1158/1078-0432.CCR-18-2293 PMC6689131

[9] Xu C, Xu Z, Zhang Y, Evert M, Calvisi DF, Chen X. beta-Catenin signaling in hepatocellular carcinoma. J Clin Invest. 2022;132(4). 10.1172/JCI154515 PMC8843739 35166233

[10] Calderaro J, Couchy G, Imbeaud S, Histological subtypes of hepatocellular carcinoma are related to gene mutations and molecular tumour classification. J Hepatol. 2017;67(4):727–738. 28532995 10.1016/j.jhep.2017.05.014

[11] Rebouissou S, Franconi A, Calderaro J, Genotype-phenotype correlation of CTNNB1 mutations reveals different ss-catenin activity associated with liver tumor progression. Hepatology. 2016;64(6):2047–2061. 27177928 10.1002/hep.28638

[12] Schulze K, Imbeaud S, Letouze E, Exome sequencing of hepatocellular carcinomas identifies new mutational signatures and potential therapeutic targets. Nat Genet. 2015;47(5):505–511. 25822088 10.1038/ng.3252 PMC4587544

[13] Amit S, Hatzubai A, Birman Y, Axin-mediated CKI phosphorylation of beta-catenin at Ser 45: a molecular switch for the Wnt pathway. Genes Dev. 2002;16(9):1066–1076. 12000790 10.1101/gad.230302 PMC186245

[14] Provost E, McCabe A, Stern J, Lizardi I, D’Aquila TG, Rimm DL. Functional correlates of mutation of the Asp32 and Gly34 residues of beta-catenin. Oncogene. 2005;24(16):2667–2676. 15829978 10.1038/sj.onc.1208346

[15] Bioulac-Sage P, Rebouissou S, Thomas C, Hepatocellular adenoma subtype classification using molecular markers and immunohistochemistry. Hepatology. 2007;46(3):740–748. 17663417 10.1002/hep.21743

[16] Dai W, Shen J, Yan J, Glutamine synthetase limits beta-catenin-mutated liver cancer growth by maintaining nitrogen homeostasis and suppressing mTORC1. J Clin Invest. 2022;132(24). 10.1172/JCI161408 PMC9754002 36256480

[17] Liang B, Zhou Y, Qian M, TBX3 functions as a tumor suppressor downstream of activated CTNNB1 mutants during hepatocarcinogenesis. J Hepatol. 2021;75(1):120–131. 33577921 10.1016/j.jhep.2021.01.044 PMC8217095

[18] Hoshida Y, Nijman SM, Kobayashi M, Integrative transcriptome analysis reveals common molecular subclasses of hepatocellular carcinoma. Cancer Res. 2009;69(18):7385–7392. 19723656 10.1158/0008-5472.CAN-09-1089 PMC3549578

[19] Boyault S, Rickman DS, de Reynies A, Transcriptome classification of HCC is related to gene alterations and to new therapeutic targets. Hepatology. 2007;45(1):42–52. 17187432 10.1002/hep.21467

[20] Tao J, Zhang R, Singh S, Targeting beta-catenin in hepatocellular cancers induced by coexpression of mutant beta-catenin and K-Ras in mice. Hepatology. 2017;65(5):1581–1599. 27981621 10.1002/hep.28975 PMC5397318

[21] Adebayo Michael AO, Ko S, Tao J, Inhibiting Glutamine-Dependent mTORC1 Activation Ameliorates Liver Cancers Driven by beta-Catenin Mutations. Cell Metab. 2019;29(5):1135–1150 e1136. 30713111 10.1016/j.cmet.2019.01.002 PMC6506359

[22] Delgado E, Okabe H, Preziosi M, Complete response of Ctnnb1-mutated tumours to beta-catenin suppression by locked nucleic acid antisense in a mouse hepatocarcinogenesis model. J Hepatol. 2015;62(2):380–387. 25457204 10.1016/j.jhep.2014.10.021 PMC4300253

[23] Ganesh S, Koser ML, Cyr WA, Direct Pharmacological Inhibition of beta-Catenin by RNA Interference in Tumors of Diverse Origin. Mol Cancer Ther. 2016;15(9):2143–2154. 27390343 10.1158/1535-7163.MCT-16-0309 PMC5010976

[24] Jadhav V, Vaishnaw A, Fitzgerald K, Maier MA. RNA interference in the era of nucleic acid therapeutics. Nat Biotechnol. 2024;42(3):394–405. 38409587 10.1038/s41587-023-02105-y

[25] Rialdi A, Duffy M, Scopton AP, WNTinib is a multi-kinase inhibitor with specificity against beta-catenin mutant hepatocellular carcinoma. Nat Cancer. 2023;4(8):1157–1175. 37537299 10.1038/s43018-023-00609-9 PMC10948969

[26] Tao J, Xu E, Zhao Y, Modeling a human hepatocellular carcinoma subset in mice through coexpression of met and point-mutant beta-catenin. Hepatology. 2016;64(5):1587–1605. 27097116 10.1002/hep.28601 PMC5073058

[27] Tao J, Krutsenko Y, Moghe A, Nuclear factor erythroid 2-related factor 2 and beta-Catenin Coactivation in Hepatocellular Cancer: Biological and Therapeutic Implications. Hepatology. 2021;74(2):741–759. 33529367 10.1002/hep.31730 PMC8326305

[28] Tan X, Behari J, Cieply B, Michalopoulos GK, Monga SP. Conditional deletion of beta-catenin reveals its role in liver growth and regeneration. Gastroenterology. 2006;131(5):1561–1572. 17101329 10.1053/j.gastro.2006.08.042

[29] Russell JO, Monga SP. Wnt/beta-Catenin Signaling in Liver Development, Homeostasis, and Pathobiology. Annu Rev Pathol. 2018;13:351–378. 29125798 10.1146/annurev-pathol-020117-044010 PMC5927358

[30] Lehrich BM, Tao J, Liu S, Development of Mutated β-catenin Gene Signature to identify CTNNB1 mutations from whole and spatial transcriptomic data in patients with HCC. JHEP Reports. 2024:101186. 39583094 10.1016/j.jhepr.2024.101186 PMC11582745

[31] Hu S, Monga SP. Wnt/-Catenin Signaling and Liver Regeneration: Circuit, Biology, and Opportunities. Gene Expr. 2021;20(3):189–199. 33472727 10.3727/105221621X16111780348794 PMC8201651

[32] Qiao Y, Wang J, Karagoz E, Axis inhibition protein 1 (Axin1) Deletion-Induced Hepatocarcinogenesis Requires Intact beta-Catenin but Not Notch Cascade in Mice. Hepatology. 2019;70(6):2003–2017. 30737831 10.1002/hep.30556 PMC7206928

[33] Goel C, Monga SP, Nejak-Bowen K. Role and Regulation of Wnt/beta-Catenin in Hepatic Perivenous Zonation and Physiological Homeostasis. Am J Pathol. 2022;192(1):4–17. 34924168 10.1016/j.ajpath.2021.09.007 PMC8747012

[34] Cappuyns S, Philips G, Vandecaveye V, PD-1(−) CD45RA(+) effector-memory CD8 T cells and CXCL10(+) macrophages are associated with response to atezolizumab plus bevacizumab in advanced hepatocellular carcinoma. Nat Commun. 2023;14(1):7825. 38030622 10.1038/s41467-023-43381-1 PMC10687033

[35] Jin S, Guerrero-Juarez CF, Zhang L, Inference and analysis of cell-cell communication using CellChat. Nat Commun. 2021;12(1):1088. 33597522 10.1038/s41467-021-21246-9 PMC7889871

[36] Guilliams M, Bonnardel J, Haest B, Spatial proteogenomics reveals distinct and evolutionarily conserved hepatic macrophage niches. Cell. 2022;185(2):379–396 e338. 35021063 10.1016/j.cell.2021.12.018 PMC8809252

[37] Liao W, Overman MJ, Boutin AT, KRAS-IRF2 Axis Drives Immune Suppression and Immune Therapy Resistance in Colorectal Cancer. Cancer Cell. 2019;35(4):559–572 e557. 30905761 10.1016/j.ccell.2019.02.008 PMC6467776

[38] Shao L, Srivastava R, Delgoffe GM, Thorne SH, Sarkar SN. An IRF2-Expressing Oncolytic Virus Changes the Susceptibility of Tumor Cells to Antitumor T cells and Promotes Tumor Clearance. Cancer Immunol Res. 2024. 10.1158/2326-6066.CIR-23-0573 PMC11150089 38517470

[39] Ruffin AT, Cillo AR, Tabib T, B cell signatures and tertiary lymphoid structures contribute to outcome in head and neck squamous cell carcinoma. Nat Commun. 2021;12(1):3349. 34099645 10.1038/s41467-021-23355-x PMC8184766

[40] Tardito S, Chiu M, Uggeri J, L-Asparaginase and inhibitors of glutamine synthetase disclose glutamine addiction of beta-catenin-mutated human hepatocellular carcinoma cells. Curr Cancer Drug Targets. 2011;11(8):929–943. 21834755 10.2174/156800911797264725

[41] Andrew C, Jason G, Yunguan W, Liver cancer initiation is dependent on metabolic zonation but decoupled from premalignant clonal expansion. bioRxiv. 2024:2024.2001.2010.575013.

[42] Senni N, Savall M, Cabrerizo Granados D, beta-catenin-activated hepatocellular carcinomas are addicted to fatty acids. Gut. 2019;68(2):322–334. 29650531 10.1136/gutjnl-2017-315448

[43] Luke JJ, Bao R, Sweis RF, Spranger S, Gajewski TF. WNT/beta-catenin Pathway Activation Correlates with Immune Exclusion across Human Cancers. Clin Cancer Res. 2019;25(10):3074–3083. 30635339 10.1158/1078-0432.CCR-18-1942 PMC6522301

[44] Spranger S, Bao R, Gajewski TF. Melanoma-intrinsic beta-catenin signalling prevents anti-tumour immunity. Nature. 2015;523(7559):231–235. 25970248 10.1038/nature14404

[45] Muthuswamy R, Berk E, Junecko BF, NF-kappaB hyperactivation in tumor tissues allows tumor-selective reprogramming of the chemokine microenvironment to enhance the recruitment of cytolytic T effector cells. Cancer Res. 2012;72(15):3735–3743. 22593190 10.1158/0008-5472.CAN-11-4136 PMC3780565

[46] Deng J, Miller SA, Wang HY, beta-catenin interacts with and inhibits NF-kappa B in human colon and breast cancer. Cancer Cell. 2002;2(4):323–334. 12398896 10.1016/s1535-6108(02)00154-x

[47] Nejak-Bowen K, Kikuchi A, Monga SP. Beta-catenin-NF-kappaB interactions in murine hepatocytes: a complex to die for. Hepatology. 2013;57(2):763–774. 22941935 10.1002/hep.26042 PMC3566301

[48] Han H. RNA Interference to Knock Down Gene Expression. Methods Mol Biol. 2018;1706:293–302. 29423805 10.1007/978-1-4939-7471-9_16 PMC6743327

[49] Rakhra K, Bachireddy P, Zabuawala T, CD4(+) T cells contribute to the remodeling of the microenvironment required for sustained tumor regression upon oncogene inactivation. Cancer Cell. 2010;18(5):485–498. 21035406 10.1016/j.ccr.2010.10.002 PMC2991103

[50] Patil MA, Lee SA, Macias E, Role of cyclin D1 as a mediator of c-Met- and beta-catenin-induced hepatocarcinogenesis. Cancer Res. 2009;69(1):253–261. 19118010 10.1158/0008-5472.CAN-08-2514 PMC2628201

[51] Lustig B, Jerchow B, Sachs M, Negative feedback loop of Wnt signaling through upregulation of conductin/axin2 in colorectal and liver tumors. Mol Cell Biol. 2002;22(4):1184–1193. 11809809 10.1128/MCB.22.4.1184-1193.2002 PMC134640

[52] Wong AM, Ding X, Wong AM, Unique molecular characteristics of NAFLD-associated liver cancer accentuate beta-catenin/TNFRSF19-mediated immune evasion. J Hepatol. 2022;77(2):410–423. 35351523 10.1016/j.jhep.2022.03.015

[53] Jung YS, Park JI. Wnt signaling in cancer: therapeutic targeting of Wnt signaling beyond beta-catenin and the destruction complex. Exp Mol Med. 2020;52(2):183–191. 32037398 10.1038/s12276-020-0380-6 PMC7062731

[54] Ganesh S, Shui X, Craig KP, RNAi-Mediated beta-Catenin Inhibition Promotes T Cell Infiltration and Antitumor Activity in Combination with Immune Checkpoint Blockade. Mol Ther. 2018;26(11):2567–2579. 30274786 10.1016/j.ymthe.2018.09.005 PMC6225018

