# Supplementary material for: Precision targeting of β-catenin induces tumor reprogramming and immunity in hepatocellular cancers
Source: Res Sq. 2024 Dec 12:rs.3.rs-5494074. Preprint. [Version 1] doi: 10.21203/rs.3.rs-5494074/v1 (PMC11661417; doi:10.21203/rs.3.rs-5494074/v1)
Supplement: Supplement 1 [file NIHPPRS5494074V1-supplement-1.pdf]

Supplementary Files

This is a list of supplementary files associated with this preprint. Click to download.

- MergedOnlineSupplementFiguresLegendsMethods.pdf
- MergedOnlineSupplementFiguresLegendsMethods.pdf
